# Cellular and exosome mediated molecular defense mechanism in bovine granulosa cells exposed to oxidative stress

**DOI:** 10.1371/journal.pone.0187569

**Published:** 2017-11-08

**Authors:** Mohammed Saeed-Zidane, Lea Linden, Dessie Salilew-Wondim, Eva Held, Christiane Neuhoff, Ernst Tholen, Michael Hoelker, Karl Schellander, Dawit Tesfaye

**Affiliations:** 1 Institute of Animal Science, Department of Animal Breeding and Husbandry, University of Bonn, Bonn, Germany; 2 Teaching and Research Station Frankenforst, Faculty of Agriculture, University of Bonn, Königswinter, Germany; 3 Center of Integrated Dairy Research, University of Bonn, Bonn, Germany; University of South Alabama Mitchell Cancer Institute, UNITED STATES

## Abstract

Various environmental insults including diseases, heat and oxidative stress could lead to abnormal growth, functions and apoptosis in granulosa cells during ovarian follicle growth and oocyte maturation. Despite the fact that cells exposed to oxidative stress are responding transcriptionally, the potential release of transcripts associated with oxidative stress response into extracellular space through exosomes is not yet determined. Therefore, here we aimed to investigate the effect of oxidative stress in bovine granulosa cells in vitro on the cellular and exosome mediated defense mechanisms. Bovine granulosa cells were aspirated from ovarian follicles and cultured in DMEM/F-12 Ham culture medium supplemented with 10% exosome-depleted fetal bovine serum. In the first experiment sub-confluent cells were treated with 5 μM H_2_O_2_ for 40 min to induce oxidative stress. Thereafter, cells were subjected to ROS and mitochondrial staining, cell proliferation and cell cycle assays. Furthermore, gene and protein expression analysis were performed in H_2_O_2_-challenged versus control group 24 hr post-treatment using qRT-PCR and immune blotting or immunocytochemistry assay, respectively. Moreover, exosomes were isolated from spent media using ultracentrifugation procedure, and subsequently used for RNA isolation and qRT-PCR. In the second experiment, exosomes released by granulosa cells under oxidative stress (StressExo) or those released by granulosa cells without oxidative stress (NormalExo) were co-incubated with bovine granulosa cells in vitro to proof the potential horizontal transfer of defense molecules from exosomes to granulosa cells and investigate any phenotype changes. Exposure of bovine granulosa cells to H_2_O_2_ induced the accumulation of ROS, reduced mitochondrial activity, increased expression of Nrf2 and its downstream antioxidant genes (both mRNA and protein), altered the cell cycle transitions and induced cellular apoptosis. Granulosa cells exposed to oxidative stress released exosomes enriched with mRNA of Nrf2 and candidate antioxidants. Subsequent co-incubation of StressExo with cultured granulosa cells could alter the relative abundance of cellular oxidative stress response molecules including Nrf2 and antioxidants CAT, PRDX1 and TXN1. The present study provide evidences that granulosa cells exposed to oxidative stress conditions react to stress by activating cascades of cellular antioxidant molecules which can also be released into extracellular environment through exosomes.

## Introduction

Stress induced by environment or physiology of the animals is considered as one of the important causes of impaired fertility in the dairy cattle [1,2]. A considerable number of evidences manifested that, various environmental and physiological insults including diseases, heat and oxidative stress could lead to abnormal growth and function of granulosa cells in ovarian follicular development [3,4]. Subsequently, granulosa cells apoptosis is responsible for follicular atresia [5] and subsequently oocyte and ovarian dysfunction [6,7]. Oxidative stress is defined as imbalance between the level of intracellular ROS production including superoxide anion (O_2_^–^), hydrogen peroxide (H_2_O_2_), and hydroxyl radicals (^-^OH^.^) and their scavenger by antioxidants [8–10]. Although−OH is the most harmful free radical, H_2_O_2_ has long half-life than the other free radicals which allowed a longer reaction with all of the cellular component including DNA. Therefore, despite lower reactivity of H_2_O_2,_ its relatively longer half-life provides enough time for the molecule to move into the nucleus of the cell [11]. Despite the fact that cells exposed to oxidative stress respond transcriptionally [12–14], the role of extracellular vesicles including exosomes in mediating cell´s response to oxidative stress should be carefully ruled [15].

Direct or indirect interactions of mammalian gametes with the surrounding somatic cells including granulosa and theca cells is vital for successful folliculogenesis [16–19]. The bidirectional communication between oocyte and surrounding cells during follicular development [20] can be mediated by extracellular vesicles [21,22]. Extracellular vesicles including exosomes (30–150 nm), microvesicles (150–1500) and apoptotic bodies (500–2000 nm) are derived from plasma membrane, outward budding of plasma membrane and outward blebbing of apoptotic cell membrane, respectively [23]. Exosomes are nano-sized vesicles and a member of extracellular membrane vesicles which mediate cell-to-cell communication under various conditions [22,24,25]. Furthermore, they are able to carry different cytosolic macromolecules (mRNA, miRNA and proteins) which can be transferred to recipient cells and induced alterations in their physiological functions [21].

Exosomes are part of extracellular vesicles with a size of 30–150 nm [26,27] that released through exocytosis process to the extracellular space and found in various biological fluids [28–32]. Regardless of origin, exosomes have similar and specific surface proteins such as CD9, CD63, CD81 and Alix [33]. In fact, exosomes contain a cargo of nucleic acids (DNA, mRNA, ncRNA), proteins, lipids and other molecules [34] and play vital role in cell-to-cell communication resulting in physiological changes in recipient cells [35,36]. Various cell types have been shown to release exosomes with various diversity in quality and quantity into the extracellular space as a response to various environmental insults and different pathological conditions [15,37]. Especially exosomes released under oxidative stress conditions could mediate a signal to recipient cells that alter their defense mechanism to prevent cell death under oxidative stress conditions [15]. Therefore, extracellular vesicles have been regarded as signalosomes: multifunctional signaling complexes for regulating fundamental cellular and biological functions [23].

Therefore, we hypothesized that granulosa cells under oxidative stress respond via activation of the cellular Nrf2 and downstream antioxidant molecules and those molecules will be released into extracellular space through exosomes. To proof this hypothesis granulosa cells culture system was used as a model to investigate the response of cells to oxidative stress induced by H_2_O_2_. Results demonstrated the significant effect on granulosa cells ROS level, mitochondrial activity, proliferation, differentiation and cell cycle. Moreover, exosomes released from granulosa cells under oxidative stress conditions into extracellular space were investigated for the presence of antioxidant molecules as molecular responses to cellular stress.

## Material and methods

### Experimental design

To determine the right concentration of H_2_O_2_ that induces ROS accumulation without a deleterious effect on granulosa cells, differet doses of H_2_O_2_ (2.5, 5, 10, 20 and 50 μM) were used to treat in vtro cultured bovine granulosa cells. Depending on the various investigations including morphological evaluation, ROS staining, mitochondrial activity and cell viability assays as shown in supplemental figures, a concentration of 5 μM H_2_O_2_ was selected as moderate oxidative stress inducer in cultured granulosa cells in the present study. The first experiment was conduceted to assess the effect of oxidative stress on granulosa cell functions as well as their cellular and extracellular response with regard to antioxidant molecules. For that, bovine granulosa cells were aspirated from small follicles (3–8 mm) and cultured in DMEM/F-12 Ham culture medium supplemented with 10% exosome-depleted fetal bovine serum (System Biosciences, CA, USA). Sub-confluent cells were exposed to 5 μM H_2_O_2_ for 40 min. Twenty four hours post-treatment intracellular ROS level, mitochondrial activity, cell proliferation and the cell cycle assays were performed. Moreover, the cellular mRNA and protein expression levels were quantified. The spent media of the same cultured granulosa cells were collected for exosome isolation and subsequent analysis of transcript abundance for Nrf2 and its antioxidant downstream genes. The second experiment was carried out to elucidate the potential horizontal transfer of oxidative stress related molecular signal carried by exosomes from donor to recipient cells. For that, sub-confluent granulosa cells were co-cultured (with or without H_2_O_2_) with exosomes derived from spent media of stressed granulosa cells (StressExo) or from spent media of granulosa cells without stress (NormalExo). All phenotypic and molecular changes in recipient cells were investigated to proof the effect of exosome mediated horizontal transfer of defence molecules in recipient cells.

### Bovine granulosa cell culture

Bovine ovaries were collected from a local abattoir and transported within 1–2 hr to the lab in a thermic flask containing physiological saline solution (0.9% NaCl) at 37°C. Upon arrival, ovaries were washed 2–3 times with 37°C 0.9% NaCl, followed by rinsing in 70% warm ethanol for 30 sec and washed 3 times with 0.9% NaCl. Granulosa cells were aspirated from small growing follicles (3–8 mm diameter) using 18-gauge sterile needle and transferred into a 15 ml sterilized falcon tube containing Ca^2+^/Mg^2+^ free 1x phosphate buffer saline (PBS-CMF). The cumulus-oocyte-complexes (COC) were left to stabilize at the bottom of the tube under 37 ^o^C. The upper phase containing granulosa cells was carefully transferred into new tubes with 1x PBS-CMF and centrifuged at 750*xg* for 7 min. The granulosa cell pellets were re-suspended in 500 μl red blood cell (RBC) lysis buffer for 1 min, followed by adding 3 ml DMEM/F-12 Ham (Sigma, Germany) and centrifuged at 500*xg* for 5 min. Afterwards, the pellets were washed with DMEM/F-12 Ham culture media supplemented with 10% exosome-depleted fetal bovine serum (System Biosciences, USA), 100 IU/ml penicillin and 100 μg/ml of streptomycin (Sigma, Germany), and 100 μg/ml fungizone (Sigma, Germany). Cell viability and concentration were determined using trypan blue exclusion method. Finally, a total of 2.5 x 10^5^ live cell per well were seeded into CytoOne^®^ 24-well plate (Starlab International GmbH, Germany) in 600 μl DMEM/F-12 Ham culture media supplemented with 10% exosome free FBS and incubated at 37°C with 5% CO_2_.

### ROS detection

Intracellular ROS level was determined using 2’, 7’-dichlorofluorescin diacetate (H2DCFDA) (Life Technologies, Germany) according to manufacturer´s instructions with some modifications. Briefly, granulosa cells from each group were cultured in 96-well plate and then incubated with 50 μl of 75 μM H2DCFDA for 20 min in dark at 37°C. Afterwards, incubated cells were washed twice with (PBS-CMF) and images were captured under inverted fluorescence microscope (Leica DM IRB, Leica, Wetzlar, Germany) using a green-fluorescence filter and images were analyzed using imageJ 1.48v (National Institutes of Health, USA, http://imagej.nih.gov).

### Mitochondrial staining

Approximately 3 x 10^4^ live cell per well were cultured in 8-well slide chamber and subjected to mitochondrial activity assay. For this, cells were incubated with 200 nM of MitoTracker red dye (MitoTracker1 Red CMXRos, M7512; Invitrogen) for 30 min. After washing twice with PBS-CMF, cells were fixed with 4% paraformaldehyde overnight at 4°C. Fixed cells were mounted with Vectashield (H-1200) containing DAPI. Images were acquired under confocal microscope CLSM LSM-780 and analyzed with imageJ 1.48v (National Institutes of Health, USA, http://imagej.nih.gov).

### Cell proliferation assay

Cell Counting Kit-8 (CCK-8) (Dojindo Molecular Technology, Japan) was used in the present study to perform cell proliferation assay. Briefly, 1 x 10^4^ live cell per well were seeded in 96-well plate and manufacturer´s instructions 10 μl of CCK-8/well was added to sub-confluent cells from all experimental groups, and then incubated for 3 hr at 37 ^o^C in 5% CO_2_. The optical density (OD) of released formazan dye which an indirect indicator of the number of living cells was measured at a wave length of 450 nm using a microplate reader (BioTek Instruments Inc, Germany). The OD of wells that contain only culture media were used as blank for normalization purpose.

### Cell cycle assay

Flow cytometery was used to demonstrate cell cycle profile in granulosa cells from each group under study. For that, cultured cells were trypsinized and centrifuged at 750*xg* for 5 min followed by two times washing with PBS-CMF. Cells were counted and a minimum 1x10^6^ of live cells were fixed in 70% ice-cold ethanol overnight at 4°C. The fixed cells were then centrifuged at 1200*xg* for 5 min, and the pellets were re-suspended and washed twice with 500 μl of PBS-CMF. Thereafter, cells were stained with 50 μg/ml of propidium iodide (PI) and 50 μg/ml of RNase and kept at 37°C for 30 min. Cell cycle analysis was performed using BD LSRFortessa™ Flow cytometer (BD Biosciences). Data were analyzed using ModFit LT software (http://www.vsh.com/products/mflt/index.asp).

### Exosome isolation

Spent culture media from control and H_2_O_2_-challenged groups were collected and subjected to exosomes isolation procedure. Briefly, collected spent media were centrifuged at 300*xg* for 10 min to discard cells and 3000*xg* for 30 to remove dead cells and 10000*xg* to discard cellular debris followed by 30 min centrifugation at 30000*xg* to remove micro vesicles. After that, exosomes were isolated using ultracentrifugation at 120,000*xg* for 70 min in a Beckman SWTi55 rotor. Exosomes were washed with PBS-CMF and centrifuged once more at 120,000*xg* for 70 min. All centrifugation steps were performed at 4°C unless indicated. Finally, isolated exosomes were suspended in PBS-CMF and stored at -80°C for further applications.

### Nanoparticle tracking and electron microscopy analysis

Exosomes identity and purity were determined by immune blotting of exosomal and cellular marker proteins (CD63, Alix and CYCS). Concentration and size distribution of exosomes was performed using NanoSight NS300 following manufacturer protocols (Malvern Instruments, Malvern, UK). Briefly, 10 μl of purified exosomes were diluted in 1 ml PBS-CMF and used for five recording videos, videos were analyzed to give mean, mode, standard division and concentration of particles using NTA software. Moreover, electron microscope (Ziess EM 109, Carl Zeiss) was used for exosomes characterization, 30 μl drops of purified exosomes on parafilm were used to be absorbed by Formvar/carbon-coated grids. Five minutes later the Formvar/carbon-coated grids were washed using drops of PBS before incubation with 30 μl drops of 2% uranyl acetate. Grids were washed with drops of PBS then examined and captured under electron microscope.

### RNA extraction and cDNA synthesis

Total RNA was isolated from collected cells and isolated exosomes using the miRNeasy Mini kit (Qiagen, Hilden; Germany) according to manufacturer’s protocol including DNase digestion for removal of possible genomic DNA contamination. RNA concentration was measured using NanoDrop 8000 spectrophotometer (NanoDrop technologies). The cDNA was synthesized from total RNA using first stand cDNA synthesis kit (Thermo Fisher scientific, Germany). RNA concentration was adjusted using RNase free water and a maximum volume of 10 μl RNA from each replicate was co-incubated with 0.5 μl of 100 μM Oligo (dT)_18_ and 0.5 μl of Random Primer at 65°C for 5 min then chilled on ice for 2 min. Thereafter, 1 μl RiboLock RNase Inhibitor, 4 μl 5x Reaction Buffer, 2 μl dNTP and 2 μl RevertAid Reverse Trancriptase, were added and incubated at 25°C for 5 min, 37°C for 60 min and 70°C for 5 min then subjected to gene expression analysis.

### Quantitative RT-PCR analysis of selected candidate genes

Relative transcript abundance of oxidative stress response genes (Nrf2, Keap1, SOD1, CAT, PRDX1, HMOX1, TXN1 and NQO1) was quantified using cDNA generated from cultured granulosa cells and isolated exosomes. Moreover, cell proliferation related genes (CCDN2 and PCNA), cell differentiation related genes (CYP11A1 and STAR1) and proapoptotic (Casp3) and antiapoptotic (BCL2L1) related genes were quantified only in granulosa cells using quantitative real time PCR in Applied Biosystem® StepOnePlus™ System (Thermo Fisher Scientific, Germany), using iTaq™ Universal SYBR® Green Supermix (Bio-Rad Laboratories GmbH, Germany). The real time PCR was run using the following program: 95°C for 3 min, 40 cycles at 95°C for 15 sec, 60°C for 45 sec followed by melting curve analysis. Data were analyzed using comparative threshold cycle method (^ΔΔ^CT) using actin, beta (ACTB) and phosphate dehydrogenase (GAPDH) as internal controls for cellular mRNA and ACTB, GAPDH and 18S genes for exosomal mRNA. All Primers listed in S1 Table were designed using primer designing tool online software (http://www.ncbi.nlm.nih.gov/tools/primer-blast/).

### Western blotting

Protein expression of (ACTB, Nrf2, Keap1, CAT, StAR1, PCNA) proteins in granulosa cells and (CYCS, CD63 and Alix) proteins in isolated exosomes were performed using immunoblotting. Isolated cellular protein and exosomes from each group were boiled with 2x SDS loading buffer at 95°C for 5 min before loading on a 12% SDS-PAGE gel. After electrophoresis, proteins were transferred to nitrocellulose membranes (Protran®, Schleicher &Schuell Bioscience) using blotting apparatus adjusted to 84 mA for 55 min. Membranes were blocked at room temperature with Roti-block solution (Carl Roth GmbH) for 1 hr and then incubated over night at 4°C with primary antibody against each of the candidate cellular proteins (Santa Cruz Biotechnology Inc, Germany: mouse monoclonal β-Actin (1:500), rabbit polyclonal Nrf2 (1:200), Keap1 (1:300), StAR1 (1:350), PCNA (1:350) and 1:300 rabbit polyclonal CAT, Life span Biosciences, Inc. Germany) or exosomal marker proteins (Santa Cruz Biotechnology Inc, Germany: goat polyclonal CYCS (1:350), Alix (1:350) and 1:250 rabbit polyclonal CD63, System BioSciences, USA). Afterwards, the membranes were washed with Tween-Tris-buffer saline (TTBS) and then incubated with secondary antibody (Santa Cruz Biotechnology Inc, Germany: goat anti mouse (1:5000), goat anti rabbit (1:5000) and donkey anti goat (1:5000)) for 1 hr at room temperature. Thereafter, the membranes were incubated with equal amount of peroxide solution and luninol\enhancer at room temperature for 5 min in dark. Images were developed on ChemiDoc™ XRS+ system (Bio-Rad Laboratories GmbH, Germany).

### Immunocytochemistry

The cellular detection and localization of Nrf2 (Santa Cruz Biotechnology Inc, Germany, 1:200 polyclonal rabbit Nrf2) and CAT (1:250 polyclonal CAT, Life span Biosciences, Inc. Germany) proteins were determined using immunocytochemistry assay. For this, granulosa cells from each group were cultured in 4-well slide chamber and subjected to immunocytochemistry assay. Cells were fixed in 4% paraformaldehyde overnight at 4°C. Fixed cells were then washed 3 times with PBS-CMF and subsequently incubated with 0.3% triton for 10 min followed by blocking with 3% donkey serum for 1 hr at room temperature followed by incubation with primary antibody overnight at 4°C. Following that, cells were washed 3 times with PBS-CMF and incubated with fluorescent secondary antibody (Santa Cruz Biotechnology Inc, Germany, Alexa flour goat anti rabbit 1:350) for 3 hr at 37°C. After washing twice with PBS-CMF, the cells were mounted in mounting medium containing DAPI. Images were taken under laser scanning confocal microscope (LSM780-Carl Zeiss, Carl Zeiss GmbH; Germany) and analyzed with imageJ 1.48v (National Institutes of Health, USA, http://imagej.nih.gov).

### Exosome labeling and co-incubation with cultured granulosa cells

The uptake of exosomes by cultured granulosa cells was assessed after co-incubation using confocal microscope (LSM780-Carl Zeiss, Carl Zeiss GmbH; Germany). For this, purified exosomes were labeled using PKH67 dye (Sigma, Germany) according to the manufacturer’s instructions as described previously [22]. Briefly, exosomes were suspended in 1 ml of diluent C containing 5 μM PKH67 and incubated for 5 min. The labeling action was stopped by incubating for 1 min with an equal volume of exosome free FBS (system Biosciences, CA; USA) and then DMEM/F-12 media (Sigma, Germany) supplemented with 10% exosome free FBS was added and centrifuged at 120,000*xg*. Exosomes were washed two times with DMEM/F-12 and centrifuged at 120,000*xg*. Thereafter, exosomes were re-suspended in DMEM/F-12 media supplemented with 10% exosome free FBS. Granulosa cells were cultured in 8-well slide chamber in DMEM/F-12 media supplemented with 10% exosome free FBS and labeled exosomes were co-incubated with granulosa cells treated with or without H_2_O_2_. Cells were fixed in 4% paraformaldehyde overnight at 4°C. After washing 2 times with PBS-CMF, cells were mounted in mounting medium containing DAPI and the up-take of exosomes was confirmed under a laser scanning confocal microscope (LSM780-Carl Zeiss Carl Zeiss GmbH; Germany). Twenty four hours post-treatment cells were investigated for ROS level, mitochondrial activity, cell proliferation, cell cycle, protein analysis using immunoblotting and immunocytochemistry assays. Moreover, mRNA expression of Nrf2 and antioxidant downstream genes was performed after co-nicubation granulosa cells with exosomes from stressed cells (StressExo) or exosomes form untreated normal cells (NormalExo).

### Statistical analysis

Data were analyzed using GraphPad Prism (Version 5) and presented as mean ± SEM of four independent biological replicates. For response of granulosa cells to oxidative stress induced by H_2_O_2_ in the first experiment, statistical differences in the mean values between treatment groups were compared using a two-tailed student´s t-test. Statistical differences among means in the second experiment were analyzed using two-way analysis of variance (ANOVA) followed by multiple pair-wise comparisons using Tukey post-hoc test. Statistical significance was defined at p ≤ 0.05.

## Results

### Dose dependent effect of H_2_O_2_ on cultured bovine granulosa cells

In order to select a physiologically acceptable ROS inducer we have tested different doses of H_2_O_2_ (2.5, 5, 10, 20 and 50 μM) and subsequent morphological and physiological assessments were done to select the optimal dose of H_2_O_2_ for further studies. As shown in supplemetal file 1 morphological evaluation of granulosa cells after treatment revealed physical death of cells at concentrations beyond 5 micro molar of H_2_O_2._ Especially at doses beyond 50 micro molar of H_2_O_2_ significant proportion of cells were found dead. In addition to that the ROS signal started to increase at 5 μM H_2_O_2_ compared to 2.5 and it does not change beyond the 5 μM concentration (S2 Fig). Moreover, treatment of granulosa cells with all doses of H_2_O_2_ except 2.5 μM resulted in significant reduction in mitochondrial activity (S3 Fig). The 5 μM dose is the minimum dose which resulted in significant reduction in mitochondrial activity compared to the other higher doses. Therefore, due to an induction of significant accumulation of ROS with with moderate effect on morphology, mitochondrial activity and cell viability, a 5 μM H_2_O_2_ dose was selected to be used in the further experimental setups.

### Effect of oxidative stress on cell morphology, ROS accumulation and mitochondrial activity in bovine granulosa cells

**Fig 1:**
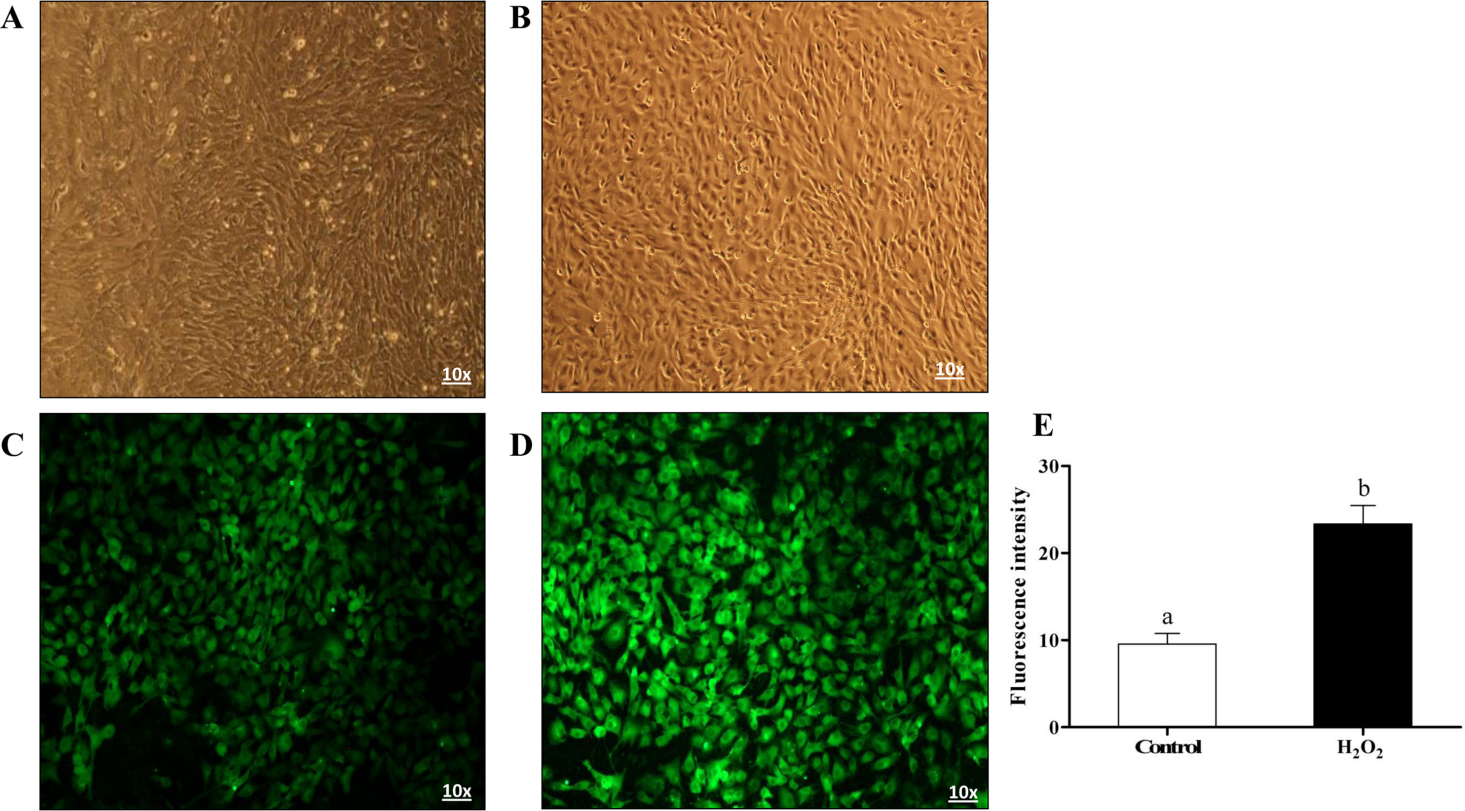
H_2_O_2_ altered cell morphology and ROS accumulation in bovine granulosa cells. Cell morphological changes (A and B), intracellular ROS level (C and D) and ROS fluorescence intensity analysis (E) in H_2_O_2_ untreated and treated groups, respectively. Data are mean ± SEM from four independent biological replicates. Bars with different letters showed statistically significant differences (*p* < 0.01).

**Fig 2:**
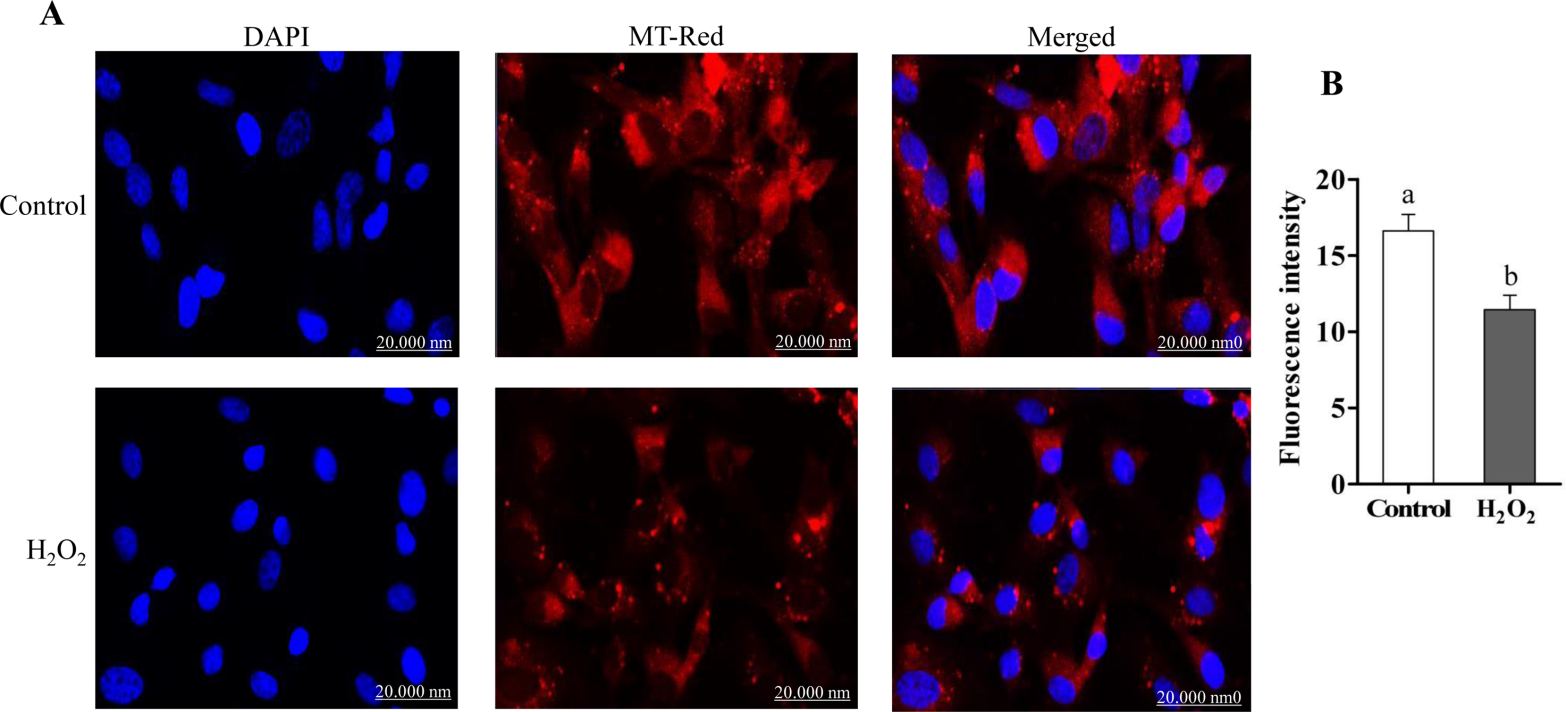
Lower level of mitochondrial activity in H_2_O_2_-treated cells compared to control group. The mitochondrial activity (A) and the fluorescence intensity analysis (B) in control and treated groups. The red colour indicated the MT-RED, while the blue colour indicated the nuclear staining using 4′,6-diamidino-2-phenylindole (DAPI). Data are mean ± SEM from four independent biological replicates. Bars with different letters showed statistically significant differences (*p* < 0.05).

The H_2_O_2_ treatment resulted in morphological changes associated with semi rounded shape and shrunken membrane (Fig 1A and 1B). Moreover, cells challenged with 5 μM H_2_O_2_ exhibited significantly higher accumulation of intracellular ROS as compared to the untreated control at 24 hours post treatment (Fig 1C and 1D). On the other hand, the mitochondrial activity of H_2_O_2_ challenged cells was lower than the untreated controls as illustrated in Fig 2.

### Oxidative stress reduced cell proliferation and G_0_/G_1_ cell cycle transition

**Fig 3:**
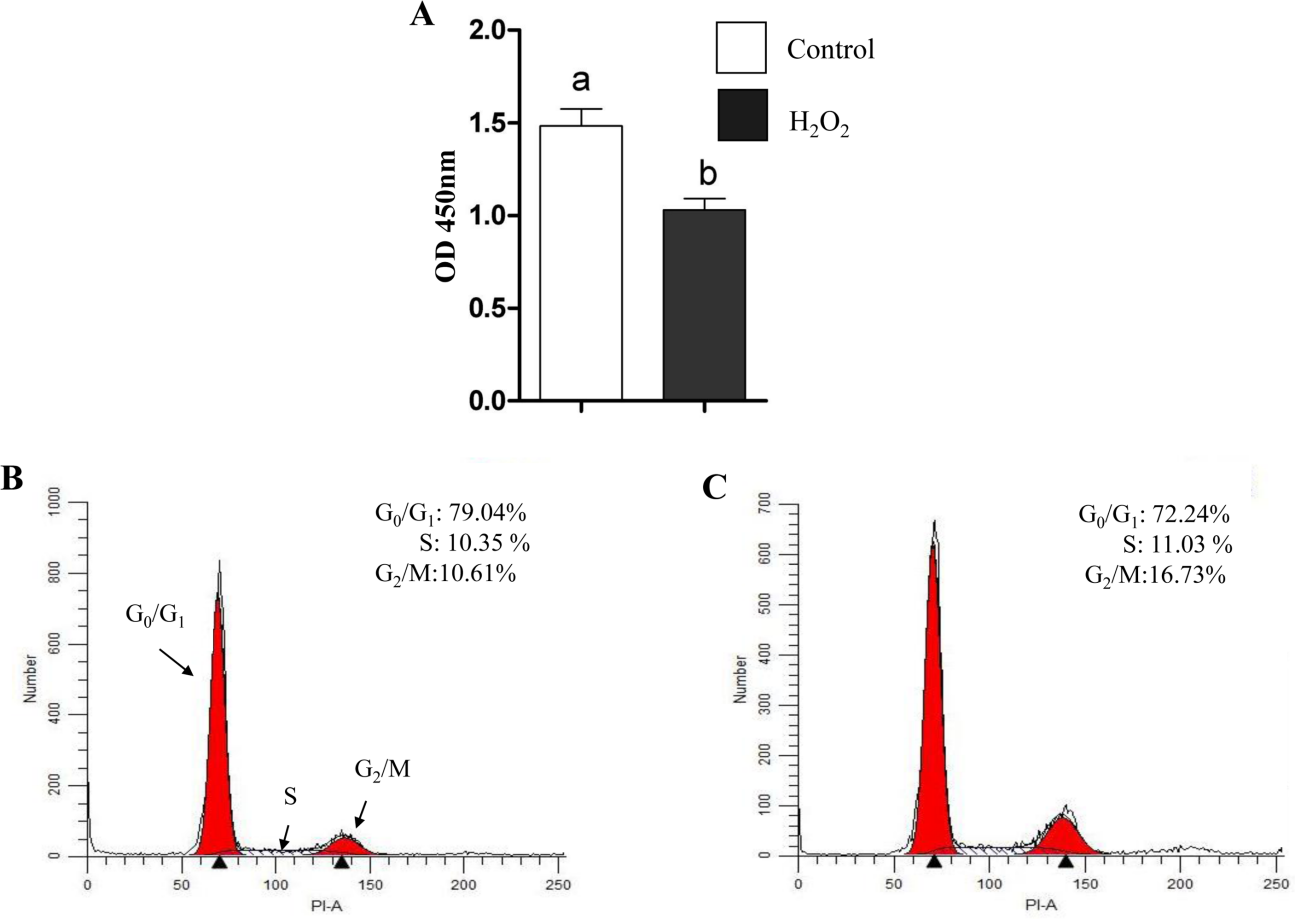
Oxidative stress resulted in reduced granulosa cell proliferation and a shift in cell cycle transition. (A) The proliferation rate in control (white bar) versus H_2_O_2_ treated cells (black bar). Cell cycle analysis of granulosa cells under normal (B) or under oxidative stress conditions (C). The Y-axis indicated the cell count, while X-axis indicated the DNA content of cells detected by PI staining. Data are mean ± SEM from four independent biological replicates. Bars with different letters showed statistically significant differences (*p* < 0.05).

Cell proliferation assay results showed a reduction in cell viability of bovine granulosa cells challenged with 5 μM H_2_O_2_ compared to untreated controls (Fig 3A). These results were in agreement with cell cycle profile, which showed lower proportion of cells arrested at G_0_/G_1_ phase (72.24%) in H_2_O_2_ challenged cells as compared to the control ones (79.04%). Moreover, the percentage of cells that were found to be arrested at the G_2_/M phase was higher in cells challenged with 5 μM H_2_O_2_ compared to untreated controls (16.73% vs. 10.61%) (Fig 3B and 3C).

### Oxidative stress increased the mRNA and protein expression levels of Nrf2 and its downstream antioxidants

**Fig 4:**
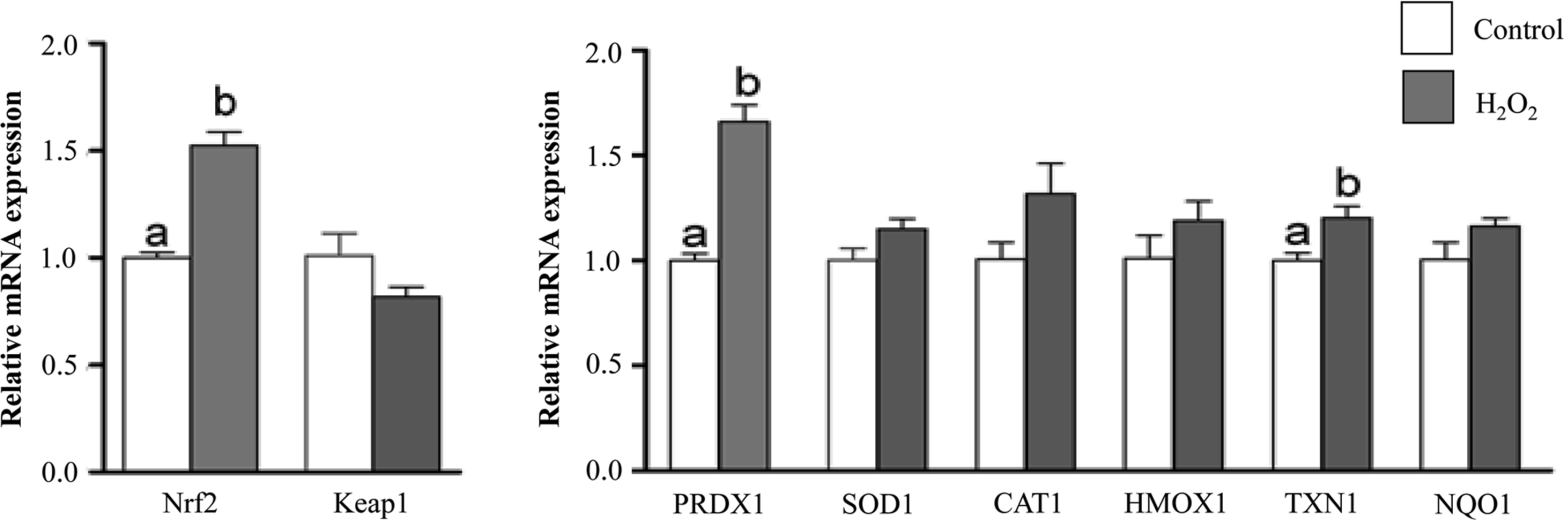
Oxidative stress increased cellular mRNA expression level of Nrf2 and its downstream antioxidants. qRT-PCR analysis of Nrf2 and downstream antioxidant genes in granulosa cells under normal (white bar) or oxidative stress (dark bar) conditions. Data are mean ± SEM from four independent biological replicates. Bars with different letters showed statistically significant differences (*p* < 0.05).

**Fig 5:**
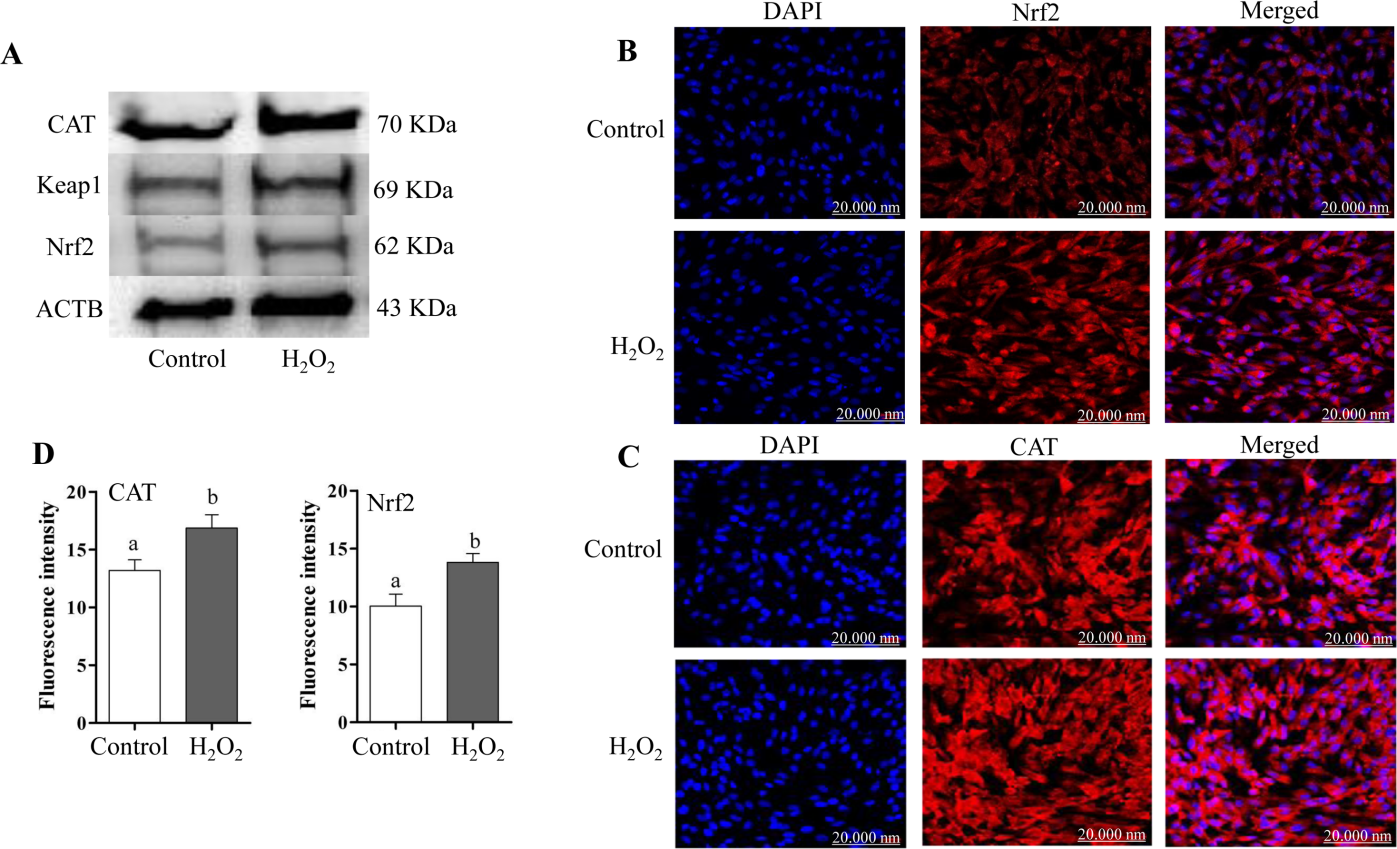
Immunoblotting analysis of Nrf2, Keap1 and CAT proteins (A) and immunocytochemistry of Nrf2 (B) and CAT (C) in bovine granulosa cells under oxidative stress and normal conditions while, (D) the fluorescence intensity analysis of Nrf2 and CAT proteins signal. The red colour indicated the expression of proteins, while the blue colour indicated the nuclear staining using 4′,6-diamidino-2-phenylindole (DAPI). Data are mean ± SEM from four independent biological replicates. Bars with different letters showed statistically significant differences (*p* < 0.05).

Significantly higher mRNA and protein expression levels of Nrf2 was accompanied by lower mRNA and protein levels of its inhibitor Keap1 in granulosa cells challenged with H_2_O_2_ compared to those cultured under normal conditions (Figs 4, 5A and 5B). Moreover, cells challenged with oxidative stress showed significantly increased expression level of Nrf2 downstream antioxidants namely: PRDX1 and TXN1. However, despite an elevated expression of mRNA for SOD1, CAT, HMOX1 and NQO1 antioxidants in challenged granulosa cells but those differences were not statistically significant (Fig 4). In contrary to that immunoblotting and immunocytochemistry assays revealed a significantly higher level of CAT protein in granulosa cells under oxidative stress conditions (Fig 5A and 5C).

### Expression of cell proliferation, differentiation, apoptosis marker genes under oxidative stress conditions

**Fig 6:**
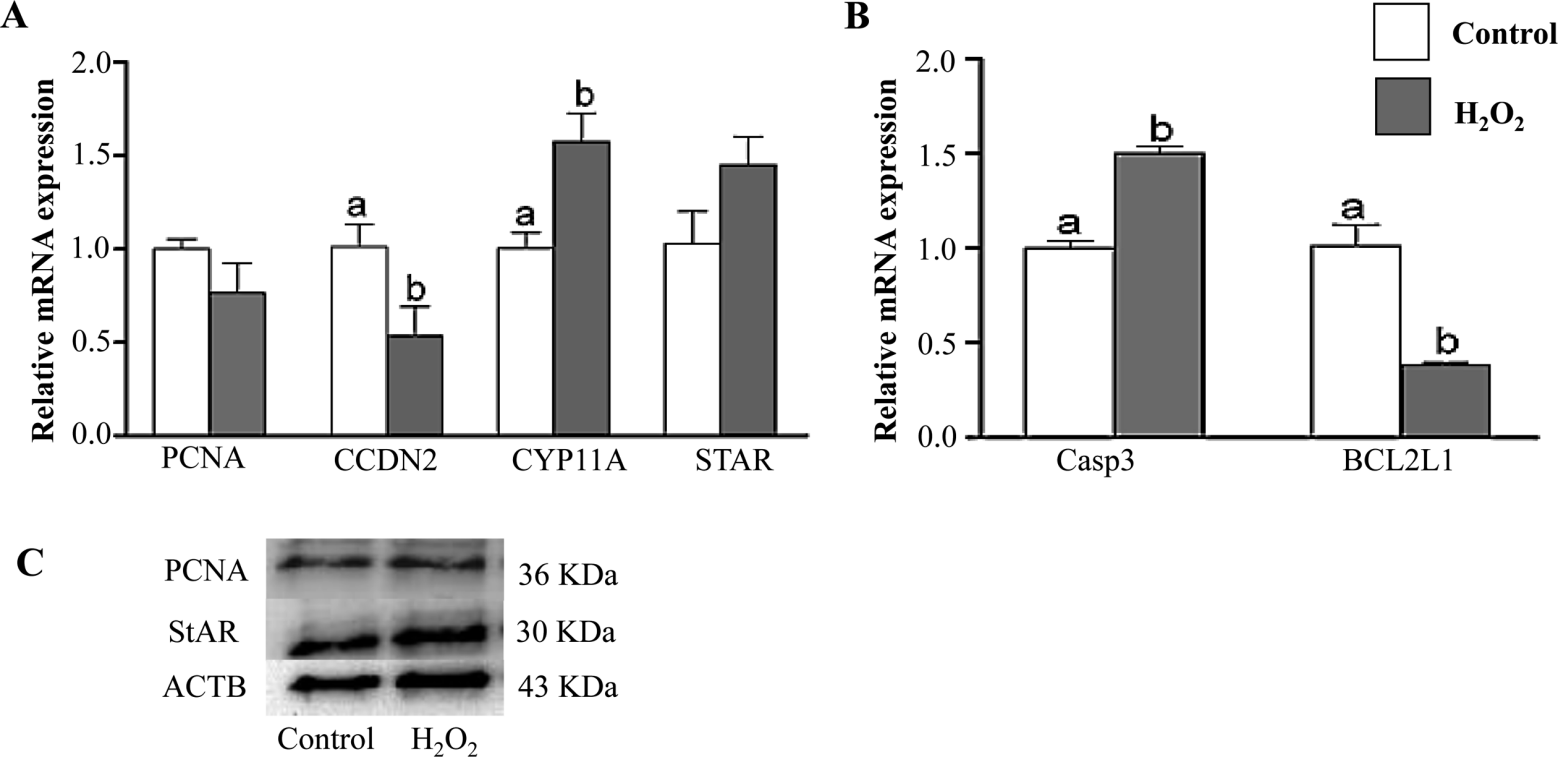
Quantitative RT-PCR analysis of cellular proliferation and differentiation marker genes (A) and pro- and anti-apoptotic marker genes (B) in granulosa cells under normal (white bar) or oxidative stress (dark bar) conditions. Western blot analysis of PCNA and StAR1 proteins (C). Data are mean ± SEM from four independent biological replicates. Bars with different letters showed statistically significant differences (*p* < 0.05).

Cell proliferation and cell cycle assay results were in consistent with the mRNA and protein expression levels of candidate marker genes. Granulosa cells under oxidative stress conditions showed significant reduction in mRNA and protein levels of cell proliferation markers (PCNA and CCDN2) and anti-apoptotic (BCL2L1) marker genes. On the other hand, the mRNA and protein expression levels of genes related to differentiation (CYP11A1 and STAR1) and pro-apoptosis (Casp3) were higher in H_2_O_2_-challenged cells as compared to the control (Fig 6A, 6B and 6C).

### Exosomes released into culture media contain mRNA of Nrf2 and antioxidants

**Fig 7:**
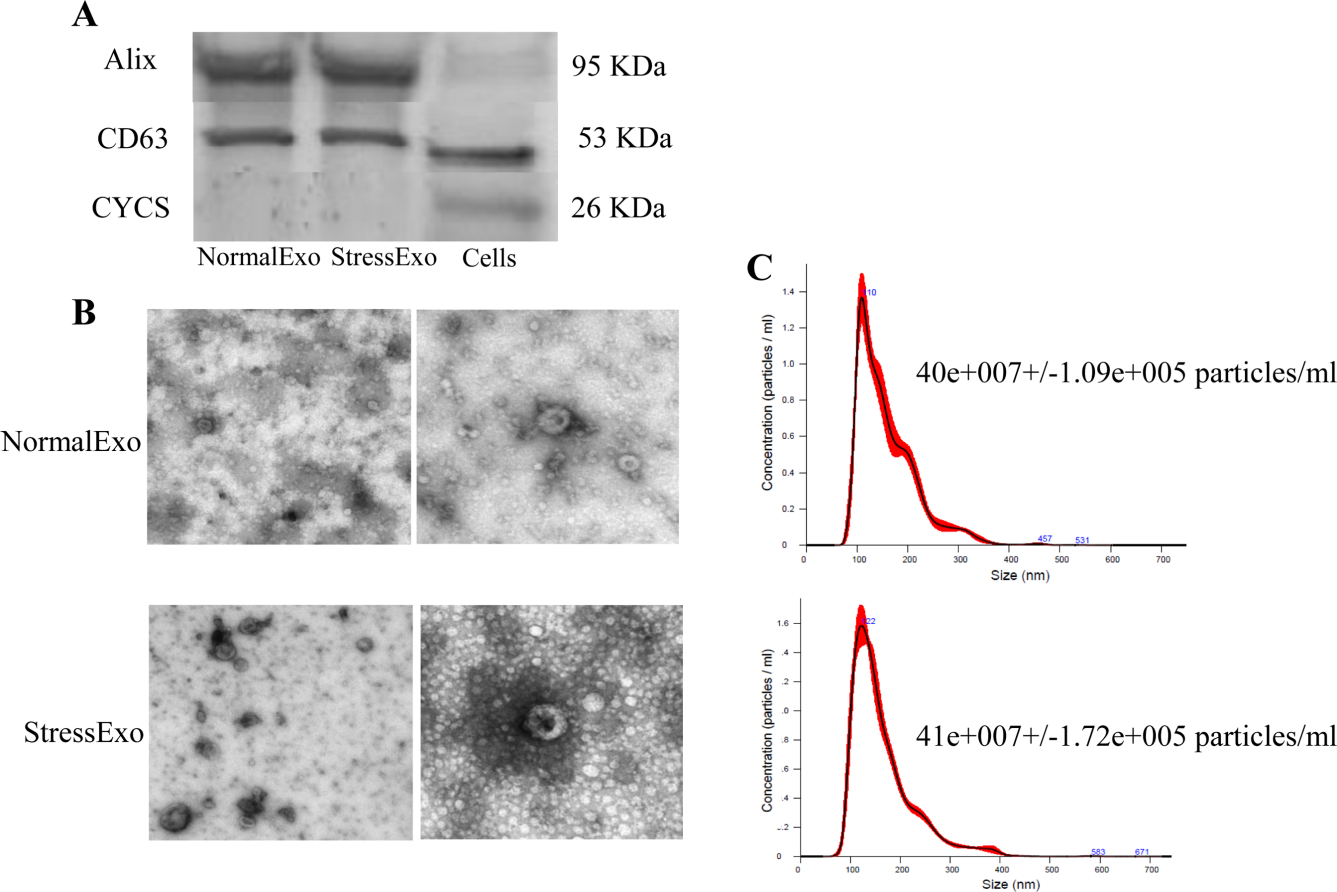
Granulosa cells exposed to oxidative stress released high concentration of exosomes with distinct size to extracellular space compared to those cultured under normal conditions. Western blot analysis of exosomes marker proteins (A). Electron microscope analysis (B) and nanoparticles tracking analysis (C) of exosomes released from granulosa cells under normal (NormalExo) and oxidative stress (StressExo) conditions {The Y-axis indicated the concentration (particles/ml) and X-axis indicated the size (nm)}.

The identity of exosomes isolated from culture supernatant was confirmed by western blot analysis of marker proteins (CD63 and Alix). The absence of any detectable protein band for cytochrome c (CYCS) confirmed the purity of exosomes isolated using ultracentrifugation technique (Fig 7A). Nanoparticle tracking results revealed that, cells released higher concentration of exosomes with distinct size to culture media when exposed to oxidative stress as illustrated in Fig 6B. Moreover, the electron microscope results confirmed the size range of the isolated exosomes using the ultracentrifugation technique (Fig 7B and 7C).

**Fig 8:**
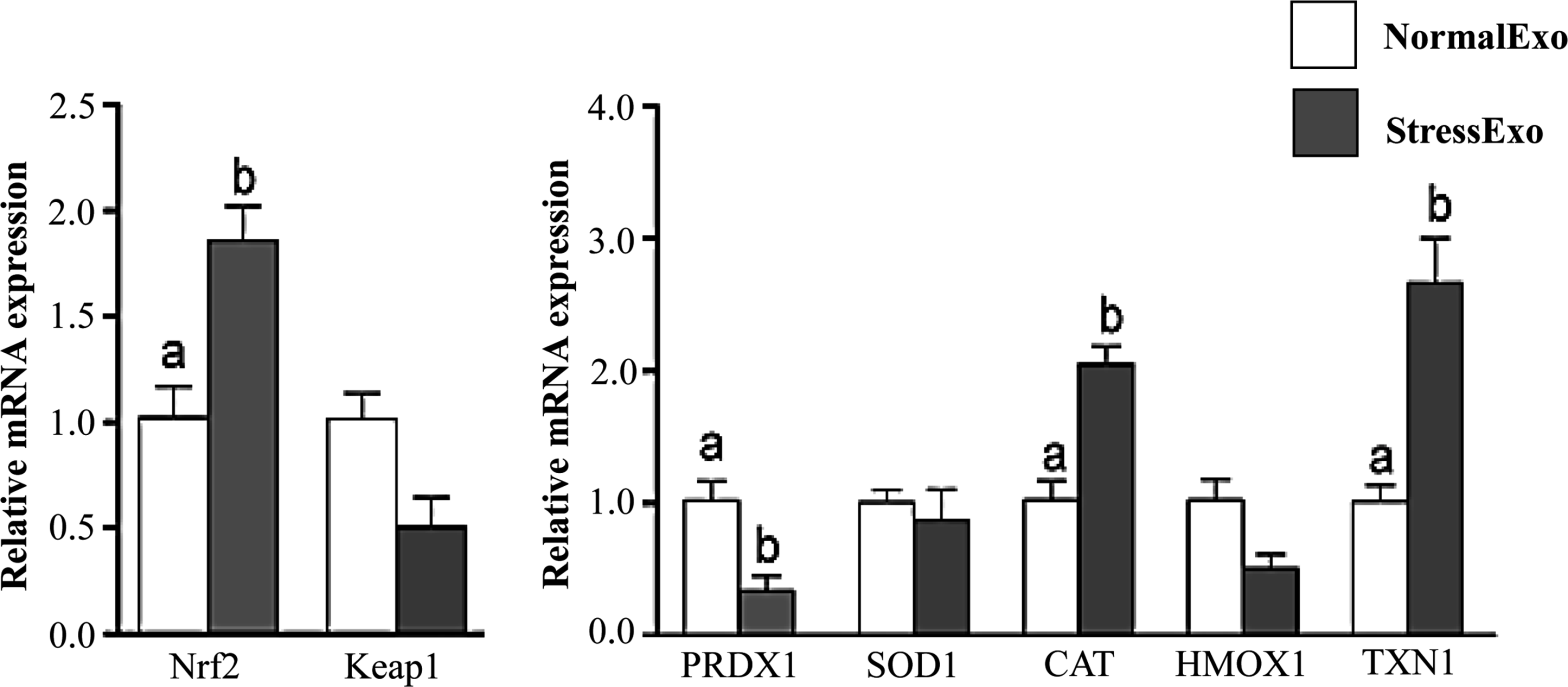
Exosomes released from granulosa cells under oxidative stress conditions contained significantly higher mRNA level of Nrf2 and antioxidant genes compared to control group. The relative mRNA expression level of Nrf2 and antioxidant genes in exosomes released from granulosa cells under normal (white bar) and oxidative stress (dark bar) conditions as analysed by qRT-PCR. Data are mean ± SEM from four independent biological replicates. Bars with different letters showed statistically significant differences (*p* < 0.05).

Exosomes released into culture media from both H_2_O_2_-treated (StressExo) and untreated granulosa cells (NormalExo) were used for RNA isolation and investigation of the abundance of Nrf2 and its downstream antioxidant genes using qRT-PCR. Exosomes released from bovine granulosa cells under oxidative stress conditions were enriched with mRNA encoded by Nrf2 and had lower level Keap1. Moreover, exosomes released under oxidative stress conditions (StressExo) contained mRNA of CAT and TXN1 and significantly lower level of PRDX1 and HMOX1 genes mRNA compared to those exosomes released by untreated cells (NormalExo). On the other hand, exosomes from both groups did not show any significant difference in their mRNA content for SOD1 gene. Interestingly, NQO1 mRNA was not detected in exosomes of both treatment groups (Fig 8).

### Horizontal transfer of oxidative stress defense mechanism in granulosa cells through exosomes

**Fig 9:**
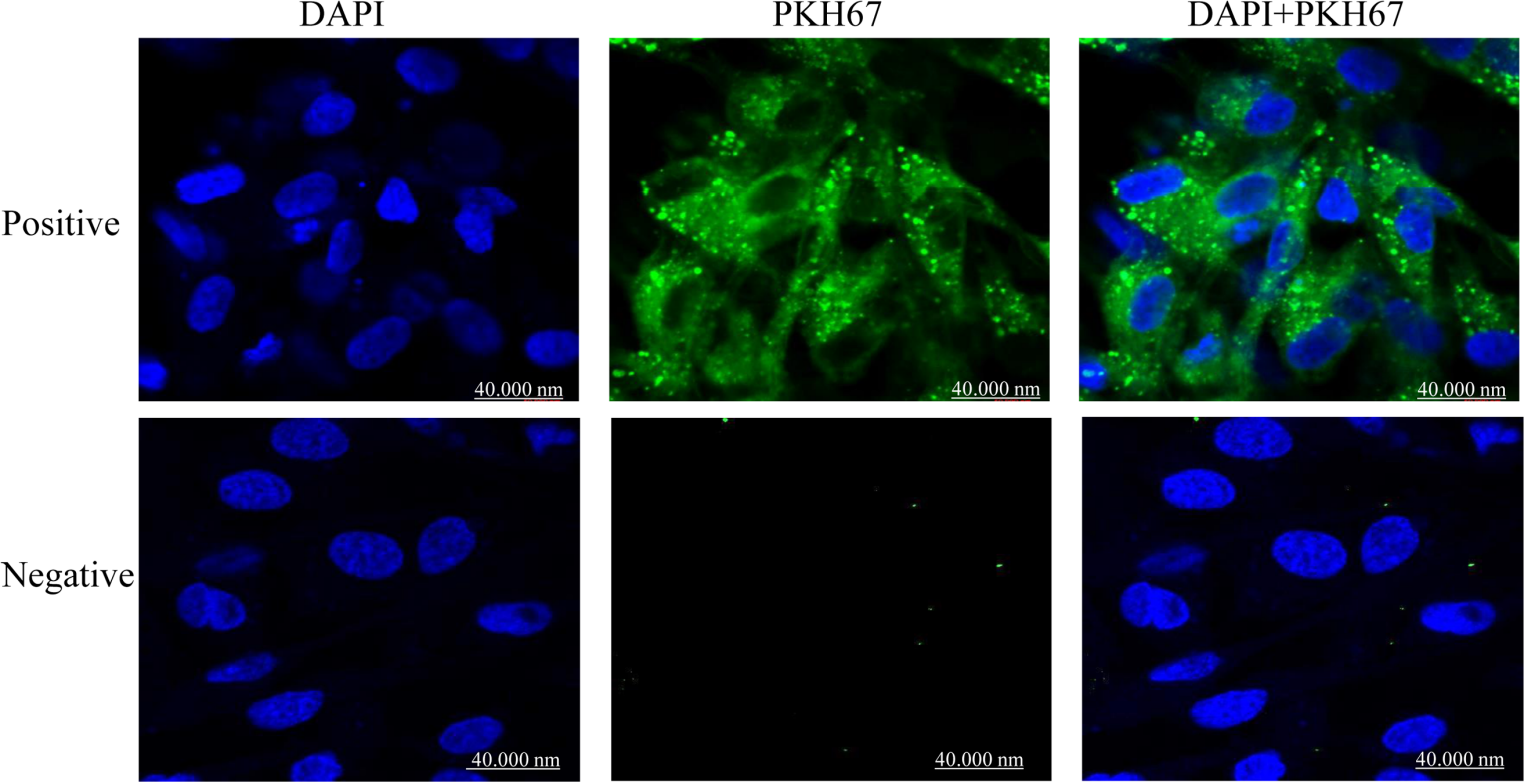
Up take of PKH67 labeled exosomes by granulosa cells after co-incubation in vitro. While green colour indicated labeled exosomes, blue colour represented nuclear staining using 4′,6-diamidino-2-phenylindole (DAPI). Images were captured under confocal microscope.

**Fig 10:**
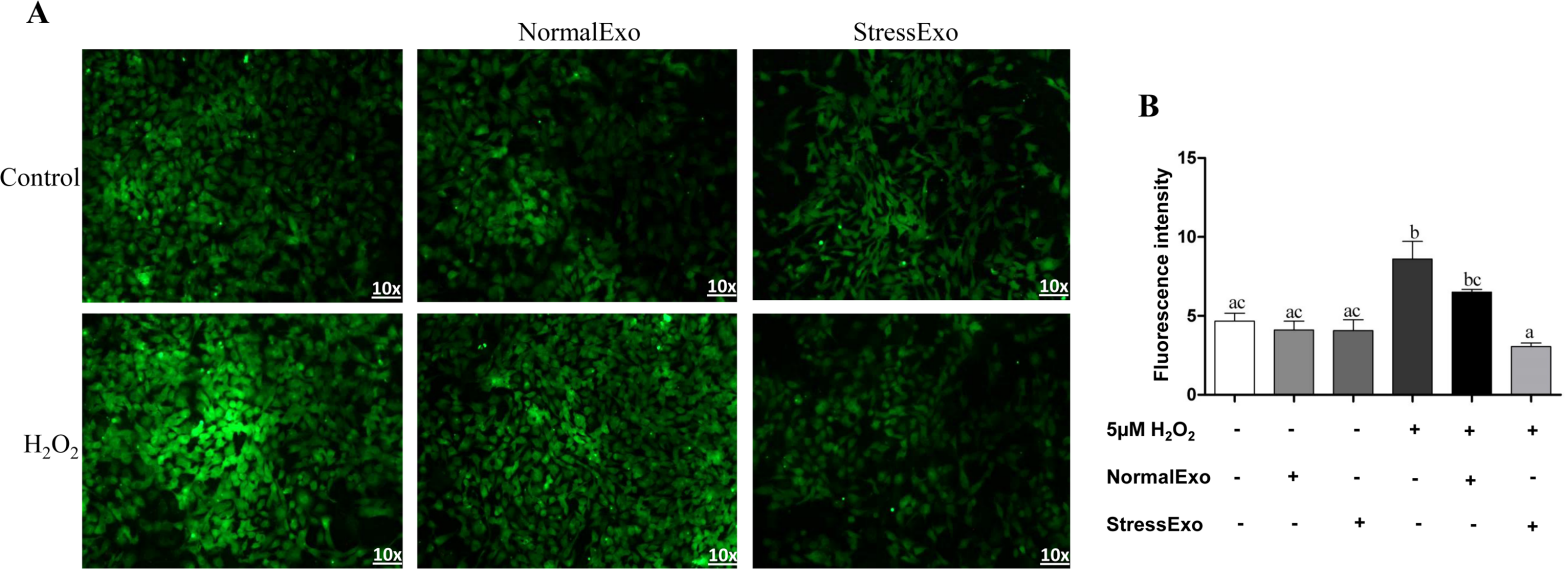
Granulosa cells co-incubated with exosomes showed reduction in ROS level under oxidative stress or normal conditions compared to control ones. The ROS level (A) was measured under normal or oxidative stress conditions in granulosa cells co-incubated with exosomes released under normal (NormalExo) or stress (StressExo) conditions compared to groups cultured without exosomes. The ROS fluorescence intensity analysis (B). Data are mean ± SEM from four independent biological replicates. Bars with different letters showed statistically significant differences (*p* < 0.05).

**Fig 11:**
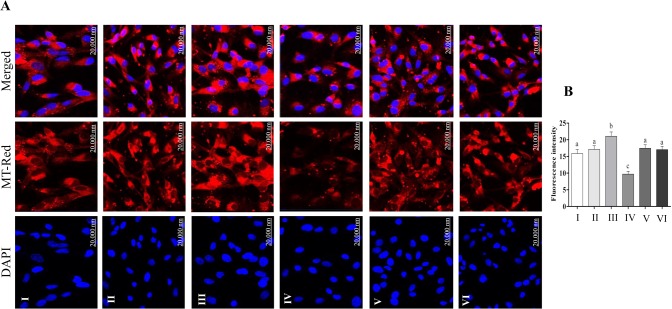
The mitochondrial activity (A) and fluorescence intensity analysis (B) in granulosa cells co-incubated with NormalExo (II), StressExo (III) compared to those not coincubated with any exosomes (I) under normal conditions and the same treatment groups under oxidative stress conditions (IV–VI). The red colour indicated the MT-RED, while the blue colour indicates the nuclear staining using 4′,6-diamidino-2-phenylindole (DAPI). Data flouresent intensity signals (B) are mean ± SEM from four independent biological replicates. Bars with different letters showed statistically significant differences (*p* < 0.05).

**Fig 12:**
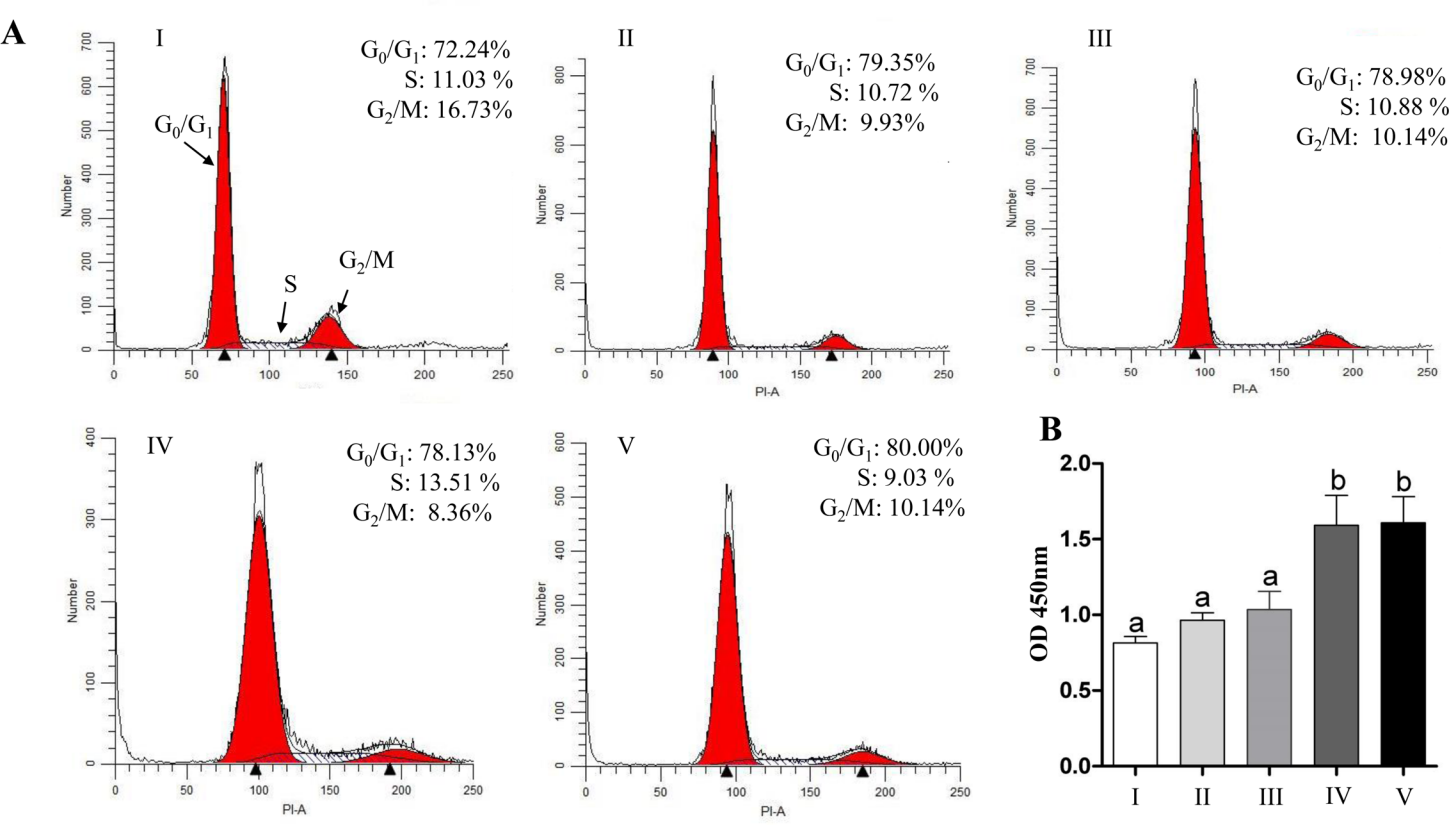
Co-incubation of bovine granulos cells with StressExo altered the cell cycle (A) and cell proliferation (B) profiles under normal or oxidative stress conditions. Granulosa cells co-incubated without exosomes (I), NormalExo (II), StressExo (III) under normal conditions or without exosomes (IV), NormalExo (V), StrssExo (VI) under oxidative stress conditions. The Y-axis indicates the cell count and X-axis indicates the DNA content of cells detected by PI staining. Data are mean ± SEM from four independent biological replicates. Bars with different letters are statistically significant (*p* < 0.05).

The uptake of labeled exosomes by granulosa cells during co-culture was confirmed under confocal microscope (Fig 9). The co-incubation of granulosa cells with StressExo resulted in lower intracellular ROS level and higher mitochondrial activity (Figs 10 and 11). Granulosa cells co-cultured under normal conditions with StressExo or NormalExo showed increased cell proliferation (Fig 12A). Similarly, cell cycle assay results demonstrated higher proportion of cells arrested at G_0_/G_1_ phase and a lower proportion in G_2_/M phase in both cells co-cultured with StressExo or NormalEXo under oxidative stress conditions (Fig 12B).

### Co-culture of granulosa cells with StressExo increased the mRNA and protein levels of Nrf2 and antioxidants

**Fig 13:**
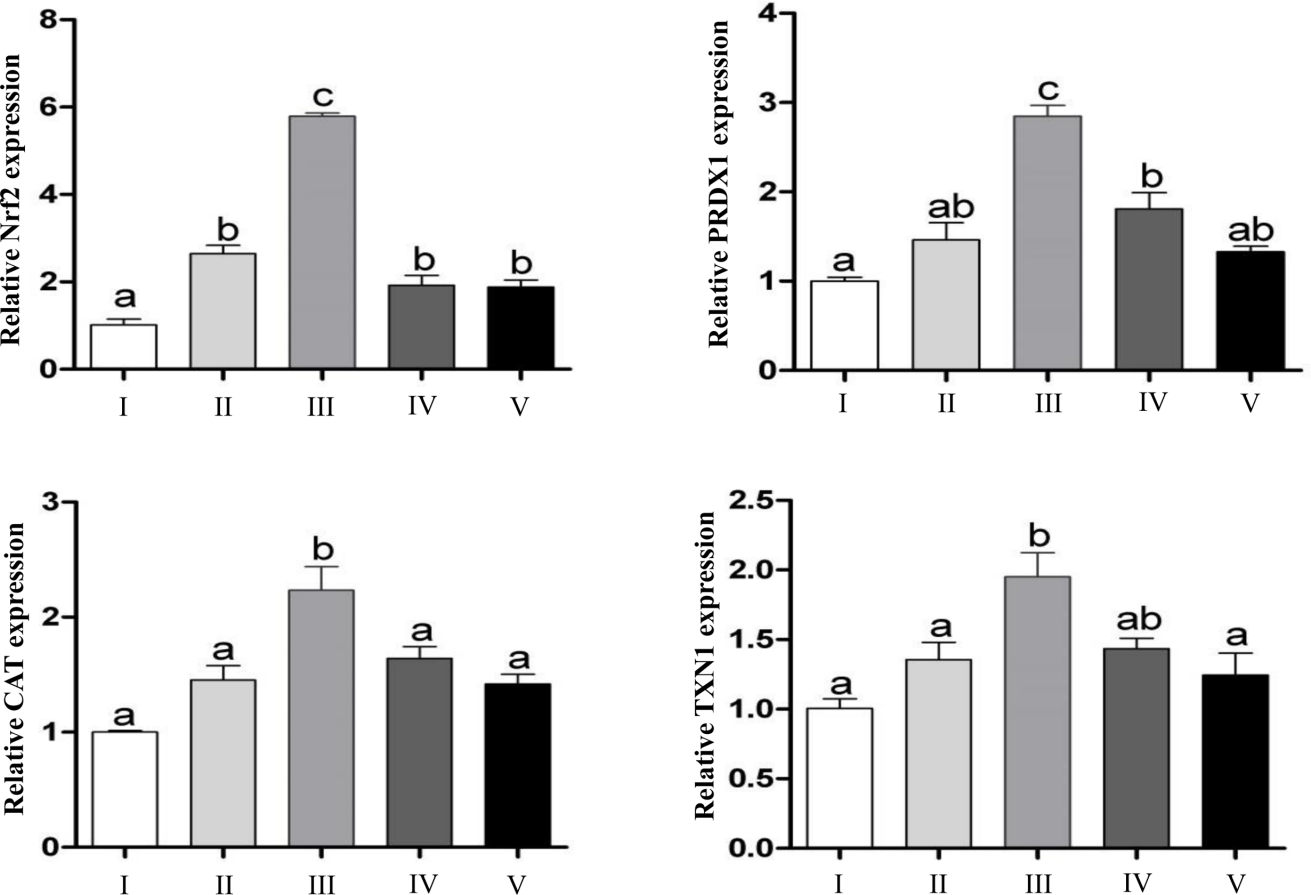
Higher mRNA expression level of Nrf2 and antioxidant genes was detected under oxidative stress conditions in granulosa cells co-incubated with StressExo. Expression level of Nrf2 and antioxidant genes in cells cultured under oxidative stress conditions (I), co-incubated with NormalExo (II) or StressExo (III) and cells cultured under normal conditions with NormalExo (IV) or StressExo (V). Data are mean ± SEM from four independent biological replicates. Bars with different letters showed statistically significant differences (*p* < 0.05).

**Fig 14:**
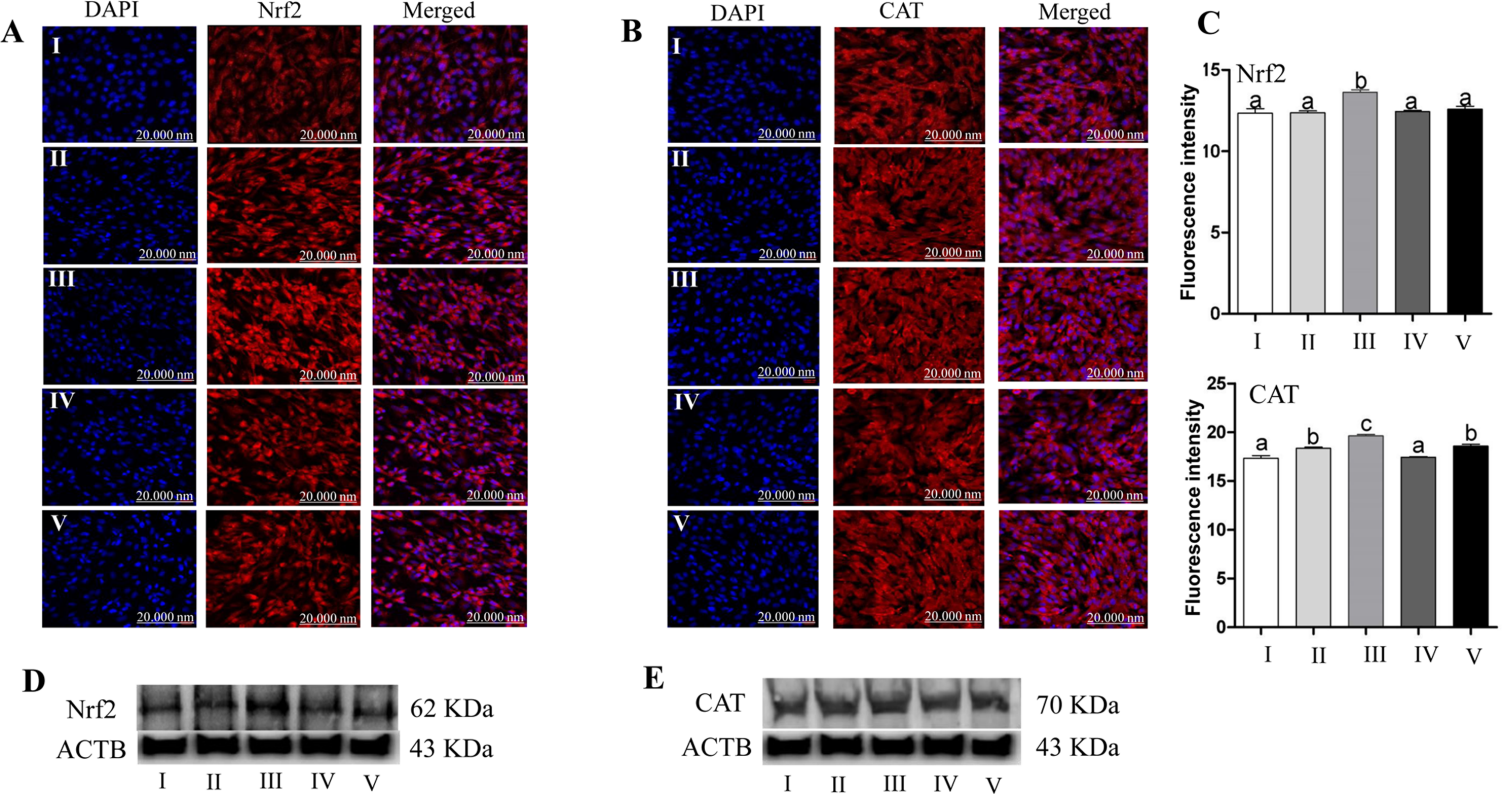
The protein expression level of Nrf2 and CAT in granulosa cells co-incubated with exosomes was in the same line with their mRNA expression level. The immunecytochemistry of Nrf2 (A) and CAT (B) in bovine granulos cells co-incubated without exosome (I), NormalExo (II) or StressExo (III) under oxidative stress conditions or under normal conditions co-incubated with NormalExo (IV) or StressExo (V). The fluorescence intensity analysis (C) of Nrf2 and CAT immunocytochemistry signals. The western blot results of all groups for Nrf2 (D) and CAT (E) proteins. In the immunocytochemstry pictures the red colour indicated the expression of proteins, while the blue colour indicated the nuclear staining using 4′,6-diamidino-2-phenylindole (DAPI). Data are mean ± SEM from four independent biological replicates. Bars with different letters are statistically significant (*p* < 0.05).

To investigate whether co-incubation of granulosa cells with exosomes result in transfer of cargo molecules especially related to of oxidative response elements. Bovine granulosa cells were co-cultured with StressExo and NormalExo either under oxidative stress or normal conditions. Quantitative RT-PCR results showed that, the mRNA expression level of Nrf2 was significantly higher in granulosa cells co-cultured with StressEXo compared to those co-cultured with NormalExo in both under oxidative stress and normal conditions. Similarly, downstream genes of Nrf2 (CAT, PRDX1 and TXN1) showed higher abundance in granulosa cells co-cultured with StressExo compared to those cocultured with NormalExo (Fig 13). Similar expression was detected for Nrf2 protein level in granulosa cells co-cultured with StressExo. Higher expression of CAT protein was found in granulosa cells co-cultured with StressExo compared to those cultured with NormalExo under normal conditions (Fig 14).

## Discussion

In dairy cattle, oxidative stress can be a result of several environmental and physiological factors such as heat stress, diet, high milk production, negative energy balance, diseases [38–42], that can lead to numerous deleterious effects on female reproduction and fertility [43,44]. Despite the fact that oxidative stress is an imbalance between reactive oxygen species (ROS) and antioxidants [9,10], large set of evidences are available for the critical physiological role of ROS particularly during folliculogenesis and ovulation being locally produced within the follicle by endothelial cells, neutrophils and macrophages [45]. However, excess ROS level including H_2_O_2_ leads to granulosa cells apoptosis and subsequently oocyte dysfunction [46]. Granulosa cells are layers of somatic cells that are surrounding oocyte and play vital role for successful folliculogenesis and subsequently embryo development and pregnancy outcome [47,48]. Granulosa cells and their antioxidant system during maturation are responsible for preventing oocyte from oxidative stress damage [49,50]. Excess ROS level in granulosa cells results apoptosis [51–53], which in turn resulted follicular atresia and ovarian dysfunction [54]. Sevral evidences are accumulated for the effect of exogenous oxidative stress induced by H_2_O_2_ resulted in higher ROS accumulation which is harmful for DNA, lipid, protein as well as mitochondria activity and integrity and subsequently lead to granulosa cell apoptosis [3,8,46,52,55]. In agreement with this, in the present study exposure of bovine granulosa cells to moderate level of H_2_O_2_ resulted in higher intracellular ROS level accompanied with lower mitochondrial activity compared to untreated control (Figs 1 and 2). Even though mitochondria are considered one of the main sources of ROS in mammalian cells [56,57], its integrity is essential for steroidogenesis in granulosa cells [52,58]. Therefore, the reduction in activity of mitochondria in stressed granulosa cells in the present study may result in disturbances in the energy metabolism of those granulosa cells and proper steriogenesis is impaired.

Cell cycle is a process regulated by growth factors that control different cellular pathways such as proliferation [59]. It has been reported that H_2_O_2_ induced G_2_/M cell cycle arrest and prevented osteoblasts cell proliferation by the reducing the expression of cyclin B1 [60]. Similarly, our results revealed that the proportion of cells under G_0_/G_1_ phase were reduced while; higher proportion of cells were arrested at G_2_/M phase in H_2_O_2_-challenged granulosa cells which is associated with a reduction in cell proliferation rate (Fig 3). This was further validated with the expression level of transcripts related to cell proliferation and cell cycle (PCNA and CCDN2). Moreover, significantly higher expression of pro-apoptotic marker gene (Casp3) with concomitant reduction of anti-apoptotic gene (BCL2L1) was observed in garnulosa cells under oxidative stress conditions as it has been reported before [46].

The mammalian cells are outfitted with an assortment of antioxidants that serve to offset the impact of oxidative stress. The harmonic expression of these antioxidant genes eliminates the stress in order to reach the homeostasis state in a manner of prohibiting damage to cellular components that are sensitive to oxidative stress [61]. Nrf2-mediated oxidative stress response is one of the most important cytoprotective mechanisms for antioxidant induction and it is sequestered in cytosol by Keap1 protein. Under oxidative stress conditions, Nrf2 is released from Keap1, translocated to nucleus, binds to antioxidant response elements (ARE) and results in releasing of antioxidant molecules [62]. We have previously shown that the activation of Nrf2 and its downstream antioxidant genes are vital for the survival of bovine embryos under suboptimal culture conditions [63,64]. Similarly, in the present study we have evidenced the induction of the mRNA and protein of Nrf2 and its downstream antioxidants (PRDX1, SOD1, CAT, HMOX1, TXN1 and NQO1) in bovine granulosa cells in response to oxidative stress. Moreover, H_2_O_2_-challenged cells showed morphological changes accompanied with semi rounded and shrunken plasma membrane (Fig 1B). Previous studies reported that H_2_O_2_ affect cell membrane permeability by altering changes in membrane composition and stage of cell cycle [65]. The exposure of osteosarcoma cells to H_2_O_2_ resulted in rounded shape and detachment of cells [66]. These findings may a result of dysregulation of focal adhesion and adhesion skeleton [67], which may result in increased distance and gaps between cells and result in impaired cell-to-cell communication.

The communication between various follicular somatic cells and the gamete during folliculogenesis can be either via gap junctions or through signal molecules mediated by the extracellular environment mainly follicular fluid. The presence and potential role of extracellular vesicles especially exosomes in follicular fluid has been reported in mare [25], women [68] and cow [22,69]. The quality and quantity of these extracellular vesicles vary depending on the physiological status of the cells, from where they are released [68,70,71] and are highly triggered by various stresses factors including diseases, heat stress as well as oxidative stress [72]. In agreement with that, our resultes demonstrated that culture media containing granulosa cells exposed to stress contained higher concentration of exosomes compared to those released by granulosa cells under normal conditions (Fig 7). Indeed, exosomes released under stress conditions are obviously differed in their RNA and protein contents compared to those released from physiologically normal cells and depending also on the type of stress the cells are exposed to [15,73]. Therefore, we were aiming at proofing the hypothesis that exosomes released from bovine granulosa cells under stress conditions may contain molecules associated with oxidative stress defense mechanism. Accordingly, exosomes released from bovine granulosa cells exposed to oxidative stress contained significant copy of mRNA molecules encoded by Nrf2 and selective antioxidant molecules into extracellular space. As it has been shown in Fig 4 the enrichment of antioxidant transcripts in exosomes released by the granulosa cells in response to oxidative stress is not valid for all antioxidants. Exosomal mRNA level of Nrf2, CAT and TXN1 was significantly higher in StressExo compared to NormalExo unlike PRDX1 which showed significantly lower mRNA level in StessExo compared to NormalExo. On the other hand, ther was no significant different for mRNA level of SOD1 and HMOX1 which tened to be lower in StressExo compared to NormalExo. Interstingly, NQO1 was not detected at mRNA level in both groups (Fig 8). The mechanism of selection and packaging of selective antioxidants into extracellular space through exosomes is so far not clear and it is a subject for future research.

There are several mechanisms for the uptake of exosomes by recipient cells including endocytosis [74,75], simple fusion [76,77] and exosomal surface ligands [78,79]. The uptake of exosomes through either of these mechanisms resulting in functional alterations in recipient cells depending on the cargo molecule they are carrying with [22,80]. In the present study, we were aiming at validating the potential horizontal transfer of exosome mediated oxidative stress defense molecules among bovine granulosa cells. Following co-incubation of StressExo with cultured granulosa cells there was a significant increase in cellular mRNA levels of Nrf2 and its selective downstream antioxidants (CAT, PRDX1 and TXN1) (Fig 13). Similarly, a significant increase in protein expression of Nrf2 and CAT1 genes was observed in granulosa cells coincubated with StressExo. Catalase (CAT) is an enzyme that can deactivate one million free radicals per second per molecule in a single cycle of catalytic reaction [81]. We have evidenced in the first experiment that alteration in relative abundance of cellular defense molecules was also accompanied by a reduction in ROS accumulation and the corresponding increase in mitochondrial activity under oxidative stress conditions (Figs 1 and 2). Simialrly, due to the horizontal transfer of oxidative stress defence molecules through exosomes, granulosa cells coculterd with StressExo showed a reduced ROS accumulation and the corresponding improved mitochondrial activity (Figs 10 and 11). Moreover, we have also evidenced that co-incubation of bovine granulosa cells with StressExo resulted in an increase in the proportion of cells under G_0_/G_1_ phase and decrease in proportion of cells at G_2_/M phase, which is associated with increased proliferation rate under oxidative stress conditions (Fig 12). Taken together, these results suggest that oxidative stress-released exosomes carry antioxidant molecules defense molecules which can be uptaken by the neighboring cells to enrich their cellular defense mechansism in order to protect themselves against oxidative stress condition induced by the unfavourable environment.

## Conclusion

The present study provides evidence that the survival of granulosa cells under oxidative stress conditions is dependent on their ability to activate their Nrf2 mediated oxidative stress response mechanisms. Moreover, these several cellular cascades of antioxidant molecules can also be released into extracellular space being coupled with exosomes which have a great potential in transfer of defense molecules form one cells to the others.

### Ethics approval and consent to participate

The study was conducted on bovine granulosa cells derived from ovaries collected from local abattoir and thus special approval of this experiment was not essential.

## Supporting information

S1 Fig Dose dependent effect of H_2_O_2_ on bovine granulosa cells morphology and cell death. (TIF)

S2 Fig ROS accumulation in bovine granulosa cells treated with different doses of H_2_O_2_. Data of ROS fluorescence intensity analysis are shown as mean ± SEM from four independent biological replicates in the graph presented. The quantification of ROS for cells treated with 50 μM H_2_O_2_ was not possible as most cells were floating due to the toxic effect of the H_2_O_2_. Bars with different letters showed statistically significant differences (*p* < 0.05).
(TIF)

S3 Fig The mitochondrial activity of bovine granulosa cells treated with different doses of H_2_O_2_. (TIF)

S4 Fig The effect of different doses of H_2_O_2_ on bovine granulosa cells proliferation. Data are mean ± SEM from four independent biological replicates. Bars with different letters are statistically significant (*p* < 0.05).
(TIF)

S1 Table The list of genes used for expression analysis and the corresponding forward and reverse primer sequences with amplicon size. (DOC)

## Abbreviations

DAPI: Complexes 4′,6-diamidino-2-phenylindole
DMEM/F-12: Dulbecco's Modified Eagle Medium/Nutrient Mixture F-12
H_2_O_2_: Hydrogen peroxide
PBS-CMF: Ca^2+^/Mg^2+^ free 1x phosphate buffer saline
ROS: Reactive oxygen species
NormalExo: Exosomes released from granulosa cells under normal conditions
StressExo: Exosomes released from granulosa cells under oxidative stress conditions
mRNA: Messenger RNA
qRT-PCR: Quantitative real time PCR ACTB: Actin beta
GAPDH: Glyceraldehyde-3-phosphate dehydrogenase
18S: 18s ribosomal RNA
Nrf2: Nuclear factor, erythroid 2 like 2
Keap1: Kelch like ECH associated protein 1
CAT: Catalase
PRDX1: Peroxiredoxin 1
SOD1: Superoxide dismutase 1
HMOX1: Heme oxygenase 1
TXN1: Thioredoxin
NQO1: NAD(P)H quinone dehydrogenase 1
PCNA: Proliferating cell nuclear antigen
STAR1: Steroidogenic acute regulatory protein
Casp3: Caspase 3, apoptosis-related cysteine peptidase
BCL2L1: BCL2 like 1
CD63: CD63 molecule
Alix: ALG-2 interacting protein X
CYCS: Cytochrome c
