# Supplementary figures and images for: Cellular and exosome mediated molecular defense mechanism in bovine granulosa cells exposed to oxidative stress
Source: PLoS One. 2017 Nov 8;12(11):e0187569. doi: 10.1371/journal.pone.0187569 (PMC5678720; doi:10.1371/journal.pone.0187569)

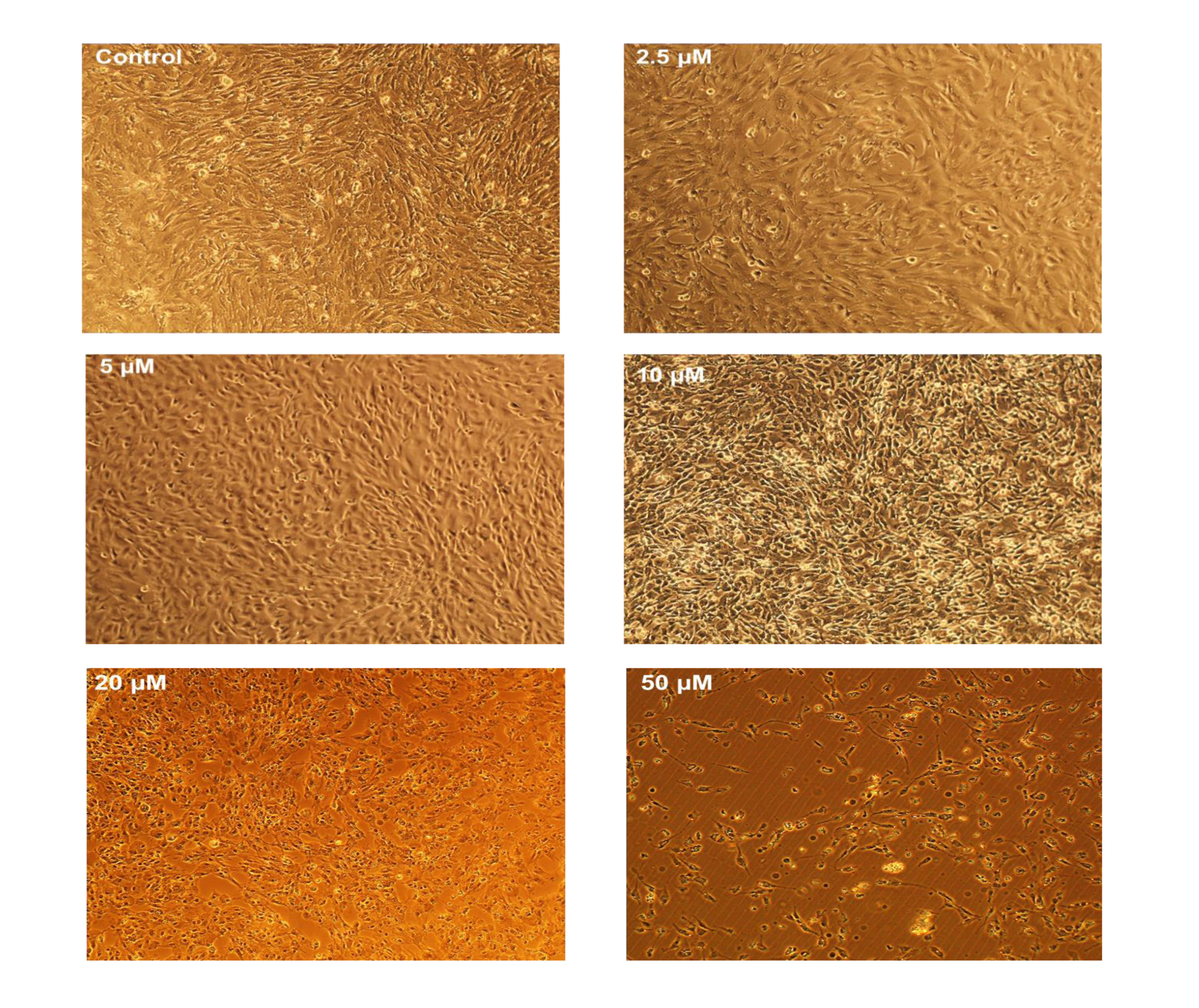

Supplement: S1 Fig — (TIF) [file pone.0187569.s001.tif]

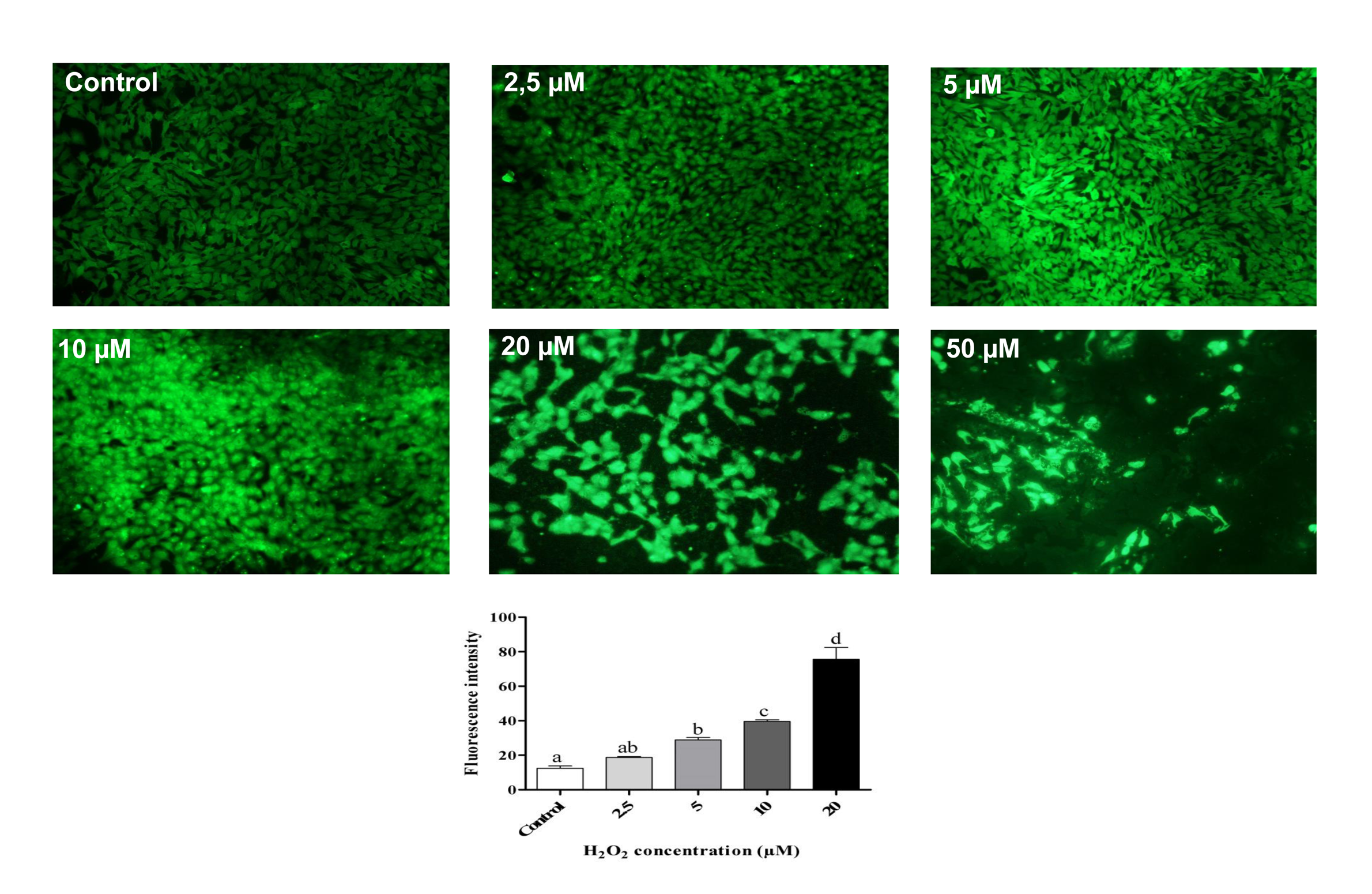

Supplement: S2 Fig — Data of ROS fluorescence intensity analysis are shown as mean ± SEM from four independent biological replicates in the graph presented. The quantification of ROS for cells treated with 50 μM H2O2 was not possible as most cells were floating due to the toxic effect of the H2O2. Bars with different letters showed statistically significant differences (p < 0.05). (TIF) [file pone.0187569.s002.tif]

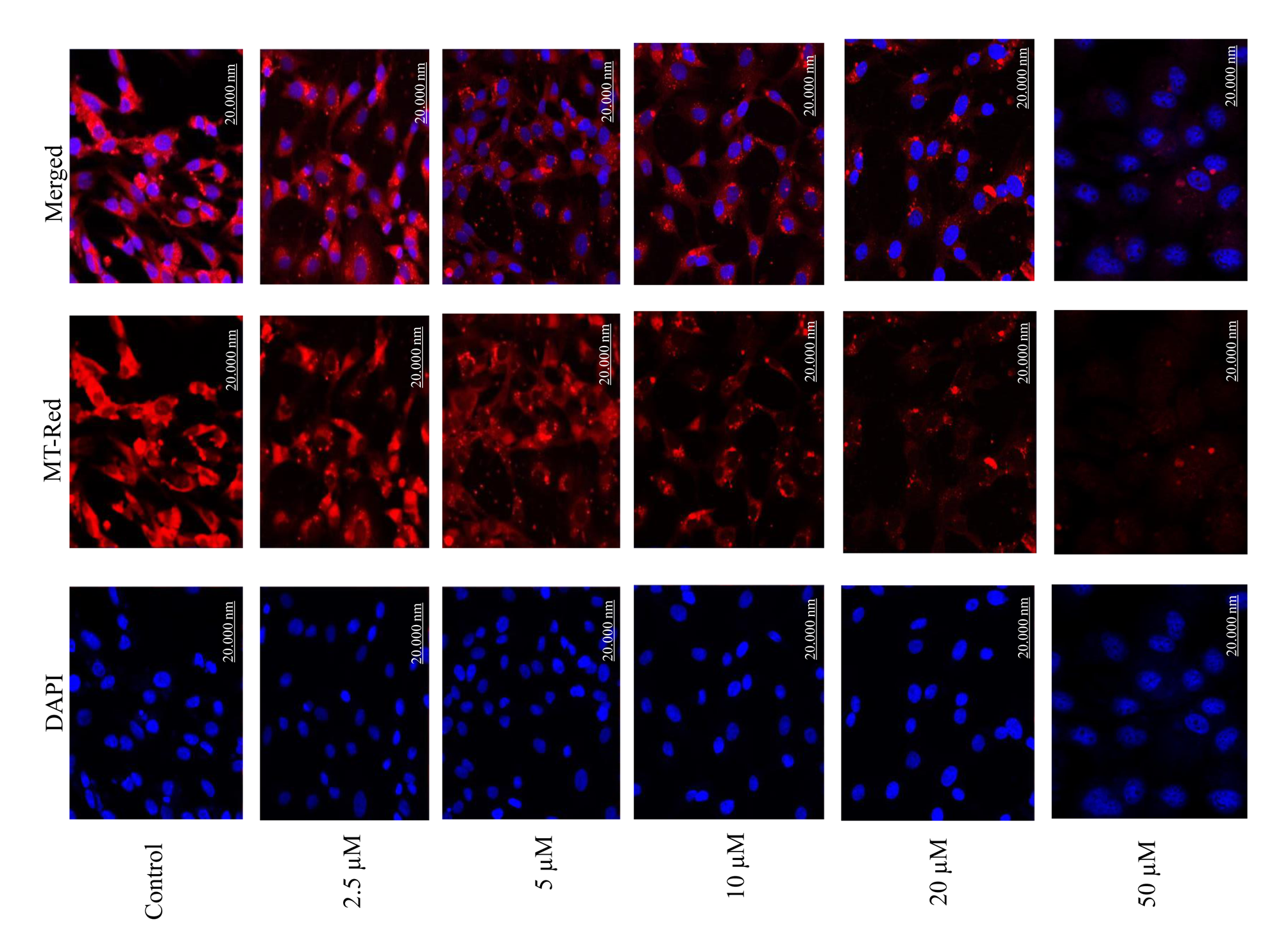

Supplement: S3 Fig — (TIF) [file pone.0187569.s003.tif]

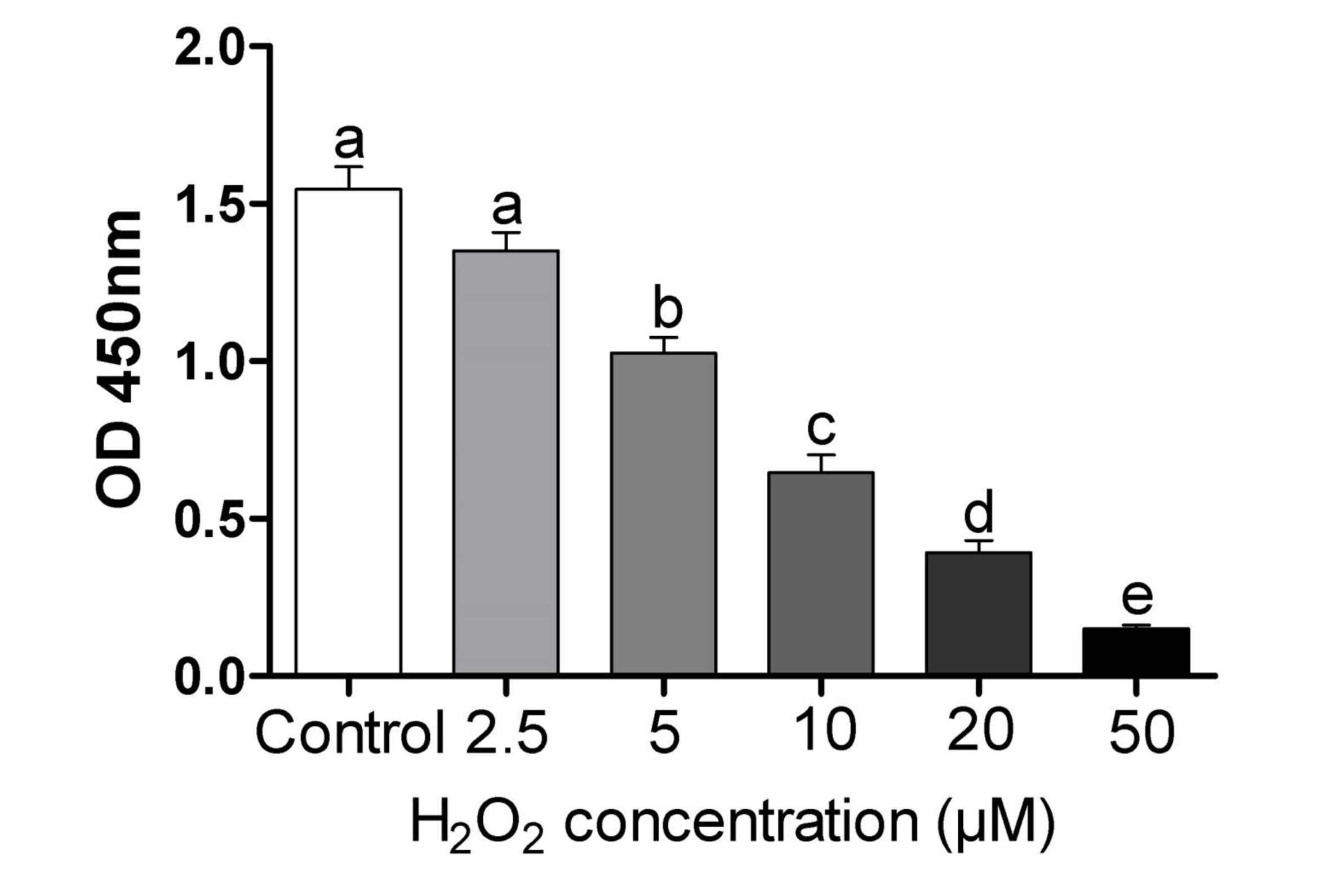

Supplement: S4 Fig — Data are mean ± SEM from four independent biological replicates. Bars with different letters are statistically significant (p < 0.05). (TIF) [file pone.0187569.s004.tif]
