# Supplementary material for: Cellular and exosome mediated molecular defense mechanism in bovine granulosa cells exposed to oxidative stress
Source: PLoS One. 2017 Nov 8;12(11):e0187569. doi: 10.1371/journal.pone.0187569 (PMC5678720; doi:10.1371/journal.pone.0187569)
Supplement: S1 Table — (DOC) [file pone.0187569.s005.doc]

| **Gene** | **Primer sequence** | **Size (bp)** | **Accession Nr** |
| --- | --- | --- | --- |
| GAPDH | F: 5´-CCCAGAATATCATCCCTGCT-3´ | 185 | NM_001034034 |
|  | R: 5´-CTGCTTCACCACCTTCTTGA-3´ |  |  |
| B-ACTIN | F: 5´-TGTCCACCTTCCAGCAGAT-3´ | 249 | NM_173979 |
|  | R: 5´-TCACCTTCACCGTTCCAGT-3´ |  |  |
| 18S | F: 5´-CGCAGCTAGGAATAATGGAA-3´ | 210 | NR_036642 |
|  | R: 5´-TCTGATCGTCTTCGAACCTC-3´ |  |  |
| NrF2 | F: 5´-CCCAGTCTTCACTGCTCCTC-3´ | 165 | NM_001011678 |
|  | R: 5´-TCAGCCAGCTTGTCATTTTG-3´ |  |  |
| Keap1 | F: 5´-TCACCAGGGAAGGATCTACG-3´ | 199 | NM_001101142.1 |
|  | R: 5´-AGCGGCTCAACAGGTACAGT-3´ |  |  |
| CAT1 | F: 5´-TGGGACCCAACTATCTCCAG-3´ | 178 | NM_001035386.1 |
|  | R: 5´-AAGTGGGTCCTGTGTTCCAG-3´ |  |  |
| PRDX1 | F: 5´-TGGATCAACACACCCAAGAA-3´ | 217 | NM_174431.1 |
|  | R: 5´-GTCTCAGCGTCTCATCCACA-3 |  |  |
| SOD1 | F: 5´-TGCCATCGTGGATATTGTAG-3´ | 189 | NM_174615 |
|  | R: 5´-GCAATTCCAATTACACCACA-3´ |  |  |
| HOMOX1 | F: 5´-CAAGGAGAACCCCGTCTACA-3´ | 225 | NM_001014912 |
|  | R: 5´-CCAGACAGGTCTCCCAGGTA-3´ |  |  |
| TXN1 | F: 5´-AGCTGCCAAGATGGTGAAAC-3´ | 215 | NM_173968.3 |
|  | R: 5´-ACTCTGCAGCAACATCCTGA-3´ |  |  |
| NQO1 | F: 5´-AACCAACAGACCAGCCAATC-3´ | 154 | NM_001034535.1 |
|  | R: 5´-CACAGTGACCTCCCATCCTT-3´ |  |  |
| Casp3 | F: 5´- TGCCACTGTATCAGGGAACA-3´ | 192 | NM_201107726 |
|  | R: 5´- TGCTCAGCACAAACATCACA-3´ |  |  |
| BCL2L1 | F: 5´-AAGTTTTCTGACCCTTTTCC-3´ | 195 | XM_015474117.1 |
|  | R: 5´-GGTCCTGGTCCTTATTTCTT-3´ |  |  |
| CCND2 | F: 5´-CGACTTCATCGAACACATCC-3´ | 279 | NM_001076372.1 |
|  | R: 5´-ATCTTTGCCAGGAGATCCAC-3´ |  |  |
| PCNA | F: 5´-CACCAGCATGTCCAAAATAC-3´ | 192 | NM_001034494.1 |
|  | R: 5´-CTGAGATCTCGGCATATACG-3´ |  |  |
| CYP11A1 | F: 5´-CGGAAAGTTTGTAGGGGACA-3´ | 177 | NM_176644.2 |
|  | R: 5´-ACGTTGAGCAGAGGGACACT-3´ |  |  |
| STAR | F: 5´-AAATCCCTTTCCAAGGTCTG-3´ | 204 | XM_006054485.1 |
|  | R: 5´-ACCAGCATTTCTGCTACTGC-3´ |  |  |
